# Synthesis of Novel Dihydropyridothienopyrimidin-4,9-dione Derivatives

**DOI:** 10.3390/molecules20035074

**Published:** 2015-03-19

**Authors:** Youngjae Kim, Minjoo Kim, Mooseong Park, Jinsung Tae, Du-Jong Baek, Ki Duk Park, Hyunah Choo

**Affiliations:** 1 Center for Neuro-Medicine, Korea Institute of Science and Technology, Seongbuk-gu, Seoul 136-791, Korea; E-Mails: 213501@kist.re.kr (Y.K.); yonlyk@naver.com (M.K.); h14507@kist.re.kr (M.P.); kdpark@kist.re.kr (K.D.P.); 2 Department of Chemistry, Yonsei University, Seodaemun-gu, Seoul 120-749, Korea; 3 Department of Chemistry, College of Natural Sciences, Sangmyung University, Seoul 110-743, Korea; E-Mail: djbaek@smu.ac.kr; 4 Department of Biological Chemistry, University of Science and Technology, Youseong-gu, Daejeon 305-350, Korea; E-Mail: jstae@yonsei.ac.kr

**Keywords:** dihydropyridothienopyrimidin-4,9-dione, pyridothienopyrimidine, successive ring formation

## Abstract

A novel molecular scaffold, dihydropyridothienopyrimidin-4,9-dione, was synthesized from benzylamine or *p*-methoxybenzylamine in six steps involving successive ring closure to form a fused ring system composed of dihydropyridone, thiophene and pyrimidone. The pharmacological versatility of the dihydropyridothenopyrimidin-4,9-dione scaffold was demonstrated by inhibitory activity against metabotropic glutamate receptor subtype 1 (mGluR1), which shows that the title compounds can serve as an interesting scaffold for the discovery of potential bioactive molecules for the treatment of human diseases.

## 1. Introduction

**Figure 1:**
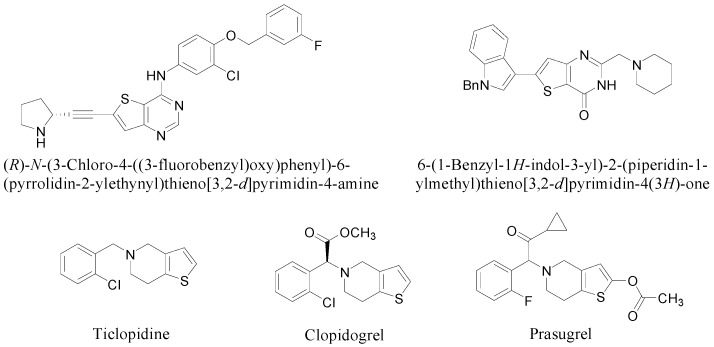
Bioactive thienopyrimidine and thienopyridine derivatives.

Much attention has been paid to the thienopyrimidine and thienopyridine scaffolds due to their notable biological and pharmacological activities (Figure 1) [1,2,3,4,5,6]. Thienopyrimidines, like (*R*)-*N*-(3-chloro-4-((3-fluorobenzyl)oxy)phenyl)-6-(pyrrolidin-2-ylethynyl)thieno[3,2-d]pyrimidin-4-amine and 6-(1-benzyl-1*H*-indol-3-yl)-2-(piperidin-1-ylmethyl)thieno[3,2-d]pyrimidin-4(3*H*)-one, show potent anticancer activity by inhibition of epidermal growth factor receptor (EGFR) [1] and vascular endothelial growth factor receptor-2 (VEGFR-2) [2], respectively. On the other hand, thienopyridine derivatives such as ticlopidine, clopidogrel, and prasugrel block P2Y_12_ receptors and thus inhibit platelet activation and aggregation [3,4,5,6].

**Figure 2:**
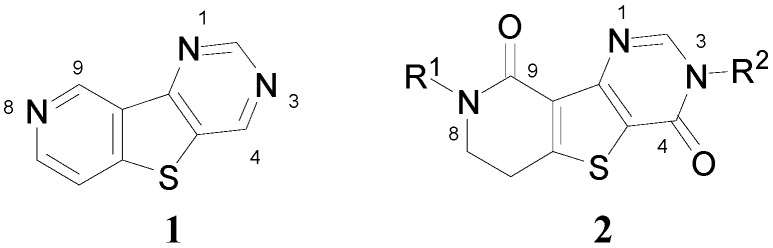
Pyridothienopyrimidine (**1**) and dihydropyridothienopyrimidin-4,9-diones **2**.

Accordingly, combining both the thienopyrimidine and thienopyridine moieties in the same molecular framework (*i.e*., pyridothienopyrimidine **1**, Figure 2) has served as an attractive strategy for designing a novel scaffold with more favorable pharmacological properties [7]. In this study, as a part of our ongoing efforts to synthesize fused tricyclic heterocycles, dihydropyridinothienopyrimidin-4,9-dione derivatives **2** were prepared starting from benzylamine or *p*-methoxybenzylamine in six steps involving successive ring formations. Also, to demonstrate the biological and pharmacological versatility, the synthesized compounds were biologically evaluated against metabotropic glutamate receptor subtype 1 (mGluR1) which is one of molecular targets for treatment of neuropathic pain.

## 2. Results and Discussion

**Scheme 1:**
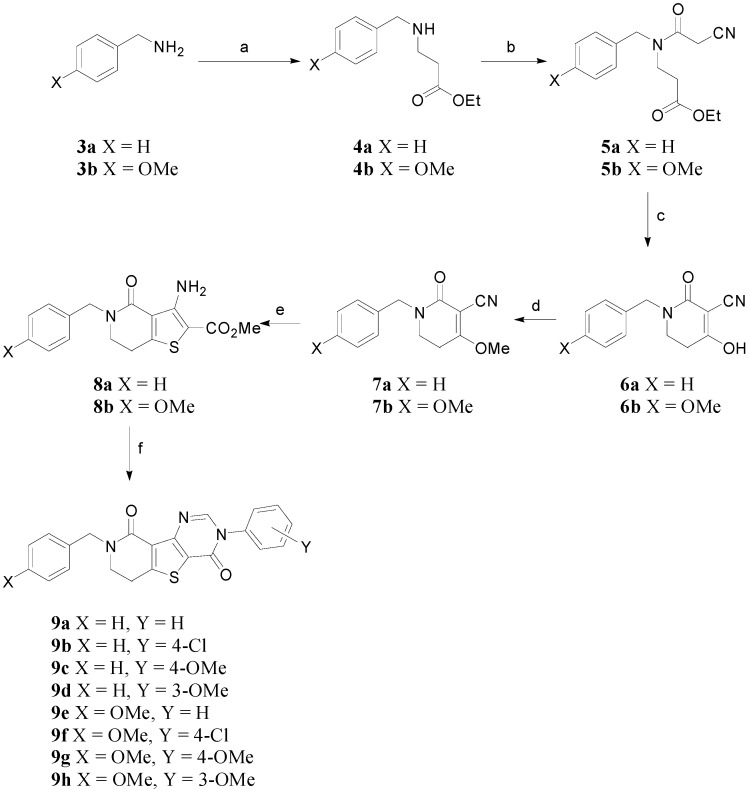
Synthesis of *N*^3^-aryl-*N*^8^-benzyldihydropyridothienopyrimidin-4,9-diones **9a**–**9h**.

To synthesize the target compounds **2**, the reaction sequence shown in Scheme 1 was used. Benzylamines **3a** and **3b** were used as starting materials. Thus, benzylamine **3a** or *p*-methoxybenzylamine **3b** were treated with ethyl acrylate in the presence of catalytic Cu(OAc)_2_ to give the corresponding β-aminoester **4a** or **4b** (47% or 57% yield, respectively). The β-aminoesters **4a** and **4b** were coupled with cyanoacetic acid using *N*,*Nʹ*-dicyclohexylcarbodimide (DCC) and 1-hydroxybenzotriazole (HOBt) in methylene chloride to afford tertiary cyanoacetamides **5a** and **5b** in 46%~63% yields.

*Reagents and conditions*: (a) ethyl acrylate, Cu(OAc)_2_, H_2_O, rt; (b) cyanoacetic acid, DCC, HOBt, DCM, 0 °C to rt; (c) Amberlyst A-26 resin, MeOH, rt; (d) Me_2_SO_4_, NaH, THF, rt to 40 °C; (e) HSCH_2_CO_2_Me, NaOMe, MeOH, 65 °C; (f) anilines, CH(OEt)_3_, AcOH, 160 °C, sealed tube.

To form the dihydropyridone ring, the cyanoacetamides **5a** and **5b** underwent modified Dieckmann condensation under acidic conditions using Amberlyst A-26 resin as catalyst to give **6a** and **6b** in 55%~64% yields [8]. The hydroxy groups of the compounds **6a** and **6b** were methylated by dimethyl sulfate and NaH (37%~63% yields). The resulting methylated compounds **7a** and **7b** were then subjected to a series of heterocyclization reactions [9,10] to give the desired dihydropyridothienopyrimidin-4,9-diones **9a**~**9h** in 20%~67% combined yields.

The synthesized compounds **9a**~**9h** were biologically evaluated against Chem-3 cells with stably expressing mGluR1 which has been considered as a potential target for treating neuropathic pain [11], and the results are summarized in Table 1. Percent inhibitions (%-inhibitions) for all the synthesized compounds were measured at 1 μM. Overall, the dihydropyridothienopyrimidin-4,9-dione derivatives showed low to moderate inhibitory activity compared with the reference compound (93.37% inhibition) [9]: the compounds **9a**, **9d**, and **9f** showed moderate inhibitory activity against mGluR1 with 23.04%, 16.54%, and 18.47% inhibition, respectively, while others showed negligible inhibitory activities.

**Table 1:** Inhibitory activities of the synthesized dihydropyridothienopyrimidin-4,9-diones **9** against mGluR1. 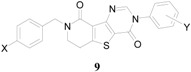

Entry	Compound	X	Y	%Inhibition at 1 μM *^a^*
1	**9a**	H	H	23.04%
2	**9b**	H	4-Cl	−1.59%
3	**9c**	H	4-OMe	3.15%
4	**9d**	H	3-OMe	16.54%
5	**9e**	OMe	H	8.29%
6	**9f**	OMe	4-Cl	18.47%
7	**9g**	OMe	4-OMe	10.35%
8	**9h**	OMe	3-OMe	11.86%
9	Reference compound *^b^*			93.37%

*^a^:* %-Inhibition of compounds at 1 μM against mGluR1; *^b^:* the reference compound is shown below.
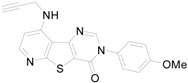

## 3. Experimental Section

### 3.1. General Methods

All reactions were carried out under dry nitrogen or argon unless otherwise indicated. Commercially available reagents were used without further purification. Solvents and gases were dried according to standard procedures. Organic solvents were evaporated with reduced pressure using a rotary evaporator. Analytical thin layer chromatography (TLC) was performed using glass plates precoated with silica gel (0.25 mm). TLC plates were visualized by exposure to UV light (UV), and then were visualized with a KMnO_4_ stain followed by brief heating on hot plate. Flash column chromatography was performed using silica gel 60 (230–400 mesh, Merck, Kenilworth, NJ, USA) with the indicated solvents. The melting points of solid products was measured by using capillary tubes for the determination of melting points (Marienfeld-Superior, Lauda-Königshofen, Germany) and an automated melting point system (OptiMelt, Stanford Research Systems, Inc. Sunnyvale, CA, USA). The tubes containing solid compounds were inserted in OptiMelt, and the temperature was slowly raised from 100 °C to 400 °C. ^1^H- and ^13^C-NMR spectra were recorded on Bruker 300 or 400 spectrometers (Bruker Co. Billerica, MA, USA). ^1^H-NMR spectra are reported as follows: chemical shift, multiplicity (s = singlet, d = doublet, t = triplet, q = quartet, m = multiplet, dd = doublet of doublet, td = triplet of doublet, brs = broad singlet, brt = broad triplet), integration, and coupling constant (*J*) in Hertz (Hz). ^1^H-NMR chemical shifts are reported relative to CDCl_3_ (7.26 ppm). ^13^C-NMR was recorded relative to the central line of CDCl_3_ (77.0 ppm). HRMS analyses were performed on Bruker compact ESI^+^ positive mode. HPLC purifications were performed on an Alliance Waters 2489 UV/Visible detector, e2695 Separations module using a Waters Xterra prep RP-18 10 μM, 10 × 250 mm column (Waters Inc. Milford, MA, USA) and a 30%–100% acetonitrile in water solvent gradient.

*Ethyl 3-(benzylamino)propanoate* (**4a**). To a solution of benzylamine **3a** (2.6 g, 24.0 mmol) in H_2_O (100 mL) were added ethyl acrylate (2 g, 20.0 mmol), and Cu(OAc)_2_ (182 mg, 1.0 mmol). The resulting mixture was stirred at room temperature for 6 h. After termination of the reaction, the solution was extracted with EtOAc, dried over MgSO_4,_ and concentrated *in vacuo*. The residue was purified by silica gel column chromatography (*n*-hexane-EtOAc = 1:1) to afford the desired product **4a** (2.34 g, 11.3 mmol, 47% yield) as a yellow oil: ^1^H-NMR (300 MHz, CDCl_3_) δ 7.36–7.23 (m, 5H), 4.14 (q, *J* = 7.2 Hz, 2H), 3.81 (s, 2H), 2.90 (t, *J* = 6.3 Hz, 2H), 2.53 (t, *J* = 6.3 Hz, 2H), 1.66 (s, 1H), 1.25 (t, *J* = 7.2 Hz, 3H); ^13^C-NMR (100 MHz, CDCl_3_) δ 172.79, 140.17, 128.42, 128.09, 126.96, 60.41, 53.77, 44.51, 34.79, 14.23; LC/MS (ESI^+^): *m/z*: calcd for C_12_H_17_NO_2_: 207.27, [M+H]^+^; found: 208.00.

*Ethyl 3-((4-methoxybenzyl)amino)propanoate* (**4b**). Following the same procedure used for the synthesis of **4a**, the reaction of (4-methoxyphenyl)methanamine **3b** (7.8 mL, 60.0 mmol), ethyl acrylate (5 g, 50.0 mmol), and Cu(OAc)_2_ (454 mg, 2.5 mmol) gave the title compound **4b** (6.72 g, 28.3 mmol, 57% yield) as a brown oil: ^1^H-NMR (400 MHz, CDCl_3_) δ 7.24–7.22 (m, 2H), 6.87–6.85 (m, 2H), 4.13 (q, *J* = 7.1 Hz, 2H), 3.79 (s, 3H), 3.74 (s, 2H), 2.88 (t, *J* = 6.4 Hz, 2H), 2.52 (t, *J* = 6.4 Hz, 2H), 1.63 (s, 1H), 1.25 (t, *J* = 7.2 Hz, 3H); ^13^C-NMR (100 MHz, CDCl_3_) δ 172.80, 158.66, 132.30, 129.26, 113.81, 60.40, 55.26, 53.16, 44.41, 34.78, 14.22; LC/MS (ESI^+^): *m/z*: calcd for C_13_H_19_NO_3_: 237.30, [M+H]^+^; found: 238.30.

*Ethyl 3-(N-benzyl-2-cyanoacetamido)propanoate* (**5a**). DCC (1.39 g, 6.75 mmol) were added to the a solution of ethyl 3-(benzylamino)propanoate **4a** (1 g, 4.82 mmol), cyanoacetic acid (492 mg, 5.79 mmol), and HOBt (782 mg, 5.79 mmol) in MC (30 mL) at 0 °C. The solution was stirred at room temperature for 12 h. After termination of the reaction, the solution was filtered. The filtrate was washed with NaHCO_3_, and brine, dried over MgSO_4_, and concentrated in vacuo. The residue was purified by silica gel column chromatography (*n*-hexane- EtOAc = 1:2) to afford the desired product **5a** (858 mg, 3.13 mmol, 65% yield) as a yellow oil: ^1^H NMR (300 MHz, CDCl_3_) δ 7.42–7.15 (m, 5H), 4.62 (d, *J* = 2.7 Hz, 2H), 4.12 (q, *J* = 7.2 Hz, 2H), 3.87 (s, 1H), 3.70 (t, *J* = 6.6 Hz, 1H), 3.54 (t, *J* = 6.3 Hz, 1H), 3.47 (s, 1H), 2.66 (t, *J* = 6.6 Hz, 1H), 2.56 (t, *J* = 6.6 Hz, 1H), 1.28–1.22 (m, 3H); ^13^C-NMR (100MHz, CDCl_3_) (isomers) δ 171.71, 171.07, 162.63, 162.57, 136.24, 135.39, 129.34, 128.86, 128.23, 127.95, 127.85, 126.08, 114.42, 113.92, 61.27, 60.81, 52.94, 48.35, 44.11, 43.00, 32.55, 32.51, 25.35, 25.23, 14.16, 14.10; LC/MS (ESI^+^): *m/z*: calcd for C_15_H_18_N_2_O_3_: 274.32, [M+H]^+^; found: 275.15.

*Ethyl 3-(2-cyano-N-(4-methoxybenzyl)acetamido)propanoate* (**5b**). Following the same procedure used for the synthesis of **5a**, the reaction of ethyl 3-((4-methoxybenzyl)amino)propanoate **4b** (6.72 g, 28.3 mmol), cyanoacetic acid (2.89 g, 34.0 mmol), DCC (8.17 g, 39.6 mmol) and HOBt (4.59 g, 34.0 mmol) in MC (125 mL) gave the title compound **5b** (3.99 g, 13.1 mmol, 46% yield) as a yellow oil: ^1^H-NMR (300 MHz, CDCl_3_) δ 7.13 (dd, *J* = 8.6, 26.1 Hz, 2H), 6.88 (dd, *J* = 8.6, 16.6 Hz, 2H), 4.55 (s, 2H), 4.12 (q, *J* = 7.1 Hz, 2H), 3.83–3.80 (m, 4H), 3.67 (t, *J* = 6.6 Hz, 1H), 3.51 (t, *J* = 6.4 Hz, 1H), 3.47 (s, 1H), 2.64 (t, *J* = 6.7 Hz, 1H), 2.56 (t, *J* = 6.4 Hz, 1H), 1.28–1.22 (m, 3H); ^13^C-NMR (100 MHz, CDCl_3_) (isomers) δ 171.70, 171.07, 162.48, 162.41, 159.53, 159.29, 129.46, 128.26, 127.52, 127.13, 114.70, 114.43, 114.22, 113.99, 61.25, 60.79, 55.37, 55.29, 52.44, 47.70, 43.81, 42.69, 32.51, 25.36, 25.24, 14.15, 14.09; LC/MS (ESI^+^): *m/z*: calcd for C_16_H_20_N_2_O_4_: 304.35, [M+H]^+^; found: 305.15.

*Benzyl-4-hydroxy-2-oxo-1,2,5,6-tetrahydropyridine-3-carbonitrile* (**6a**). To a solution of ethyl 3-(*N*-benzyl-2-cyanoacetamido)propanoate **5a** (850 mg, 3.10 mmol) in MeOH (20 mL) were added Amberlyst A-26 resin (1.5 g). The resulting mixture was stirred at room temperature for 16 h. After termination of the reaction, the resin was filtered and washed with MeOH. Then, the resin was added to the solution of TFA (2 mL) in MeOH (20 mL), and stirred at room temperature for 30 min. The mixture solution was filtered, and concentrated in vacuo to afford the desired product **6a** (390 mg, 1.71 mmol, 55% yield) as a white solid: mp 225–227 °C; ^1^H-NMR (300 MHz, DMSO-*d_6_*) δ 7.37–7.24 (m, 5H), 4.51 (s, 2H), 3.30 (t, *J* = 7.2 Hz, 2H), 2.68 (t, *J* = 6.9 Hz, 2H); ^13^C-NMR (100 MHz, DMSO-*d_6_*) δ 181.05, 163.16, 137.98, 128.97, 128.05 127.60, 115.57, 83.49, 49.35, 42.82, 29.01; LC/MS (ESI^+^): *m/z*: calcd for C_13_H_12_N_2_O_2_: 228.25, [M+H]^+^; found: 229.15.

*4-Hydroxy-1-(4-methoxybenzyl)-2-oxo-1,2,5,6-tetrahydropyridine-3-carbonitrile* (**6b**). Following the same procedure used for the synthesis of **6a**, the reaction of ethyl 3-(2-cyano-*N*-(4-methoxybenzyl)acetamido)propanoate **5b** (200 mg, 0.66 mmol), Amberlyst A-26 resin (354 mg), and TFA (2 mL) in MeOH (10 × 2 mL) gave the title compound **6b** (108.7 mg, 0.42 mmol, 64% yield) as a white solid: mp 170–173 °C; ^1^H-NMR (400 MHz, DMSO-*d_6_*) δ 7.30–7.26 (m, 2H), 7.01–6.98 (m, 2H), 4.53 (s, 2H), 3.83 (s, 3H), 3.36 (t, *J* = 7.1 Hz, 2H), 2.74 (t, *J* = 7.0 Hz, 2H); ^13^C-NMR (100 MHz, DMSO-*d_6_*) δ 180.87, 163.02, 158.95, 129.82, 129.56, 115.54, 114.37, 83.59, 55.52, 48.70, 42.53, 28.93; LC/MS (ESI^+^): *m/z*: calcd for C_14_H_14_N_2_O_3_: 258.28, [M+H]^+^; found: 259.15.

*1-Benzyl-4-methoxy-2-oxo-1,2,5,6-tetrahydropyridine-3-carbonitrile* (**7a**). To a solution of benzyl-4-hydroxy-2-oxo-1,2,5,6-tetrahydropyridine-3-carbonitrile **6a** (100 mg, 0.44 mmol) and THF (3 mL), NaH (21 mg, 0.88 mmol) was added and the reaction mixture was stirred at room temperature for 2 h. Then dimethylsulfate (0.071 mL, 0.75 mmol) was added to the mixture and the resulting solution was stirred at 40 °C for 12 h. The mixture was quenched with H_2_O and evaporated. Then it was diluted with EtOAc, extracted with EtOAc, and dried over MgSO_4_ and concentrated, the crude product was purified by silica gel column chromatography (*n*-hexane-EtOAc = 1:1) to give product **7a** (67 mg, 0.28 mmol, 63% yield) as a white solid: mp 153–157 °C; ^1^H-NMR (300 MHz, CDCl_3_) δ 7.37–7.26 (m, 5H), 4.62 (s, 2H), 4.14 (s, 3H), 3.33 (t, *J* = 6.9 Hz, 2H), 2.61 (t, *J* = 6.9 Hz, 2H); ^13^C-NMR (100 MHz, CDCl_3_) δ 177.55, 162.08, 136.66, 128.77, 128.19, 127.76, 114.15, 86.77, 58.13, 49.85, 42.07, 26.91; LC/MS (ESI^+^): *m/z*: calcd for C_14_H_14_N_2_O_2_: 242.28, [M+H]^+^; found: 243.15.

*4-Methoxy-1-(4-methoxybenzyl)-2-oxo-1,2,5,6-tetrahydropyridine-3-carbonitrile* (**7b**). Following the same procedure used for the synthesis of **7a**, the reaction of 4-hydroxy-1-(4-methoxybenzyl)-2-oxo-1,2,5,6-tetrahydropyridine-3-carbonitrile **6b** (1.74 g, 6.70 mmol), NaH (321 mg, 13.4 mmol), and dimethylsulfate (1.1 ml, 11.4 mmol) gave the title compound **7b** (677 mg, 2.45 mmol, 37% yield) as a white solid: mp 145–148 °C; ^1^H-NMR (300 MHz, CDCl_3_) δ 7.23 (d, *J* = 8.1 Hz, 2H), 6.88 (d, *J* = 8.4 Hz, 2H), 4.57 (s, 2H), 4.14 (s, 3H), 3.82 (s, 3H), 3.35 (t, *J* = 6.7 Hz, 2H), 2.65 (t, *J* = 6.7 Hz, 2H); ^13^C-NMR (75 MHz, CDCl_3_) δ 177.20, 161.95, 159.28, 129.62, 128.73, 114.16, 86.85, 58.14, 55.31, 49.29, 41.87, 27.10; LC/MS (ESI^+^): *m/z*: calcd for C_15_H_16_N_2_O_3_: 272.30, [M+H]^+^; found: 273.15.

*Methyl 3-amino-5-benzyl-4-oxo-4,5,6,7-tetrahydrothieno*[3,2-c]*pyridine-2-carboxylate* (**8a**). NaOMe (5M in MeOH, 36.0 mL, 17.9 mmol) in MeOH followed by methyl thioglycolate (1.8 mL, 20.5 mmol) were added to 1-benzyl-4-methoxy-2-oxo-1,2,5,6-tetrahydropyridine-3-carbonitrile **7a** (3.1 g, 12.8 mmol). The reaction mixture was stirred and refluxed at 65 °C for 12 h. After the reaction, the mixture was filtered using Celite and washed with MC. The crude compound was then purified by by silica gel column chromatography (*n*-hexane-EtOAc = 4:1) to give white solid product **8a** (2.44 g, 7.38 mmol, 58% yield) as a yellowish solid: mp 110–112 °C; ^1^H-NMR (300 MHz, CDCl_3_) δ 7.37–7.27 (m, 5H), 6.91 (brs, 2H), 4.70 (s, 2H), 3.82 (s, 3H), 3.53 (t, *J* = 6.9 Hz, 2H), 2.93 (t, *J* = 6.9 Hz, 2H); ^13^C-NMR (100 MHz, CDCl_3_) δ 164.55, 162.73, 153.78, 151.00, 137.12, 128.77, 127.99, 127.63, 119.74, 96.83, 51.16, 49.03, 45.82, 25.00; LC/MS (ESI^+^): *m/z*: calcd for C_16_H_16_N_2_O_3_S: 316.38, [M+H]^+^; found: 317.05.

*Methyl 3-amino-5-(4-methoxybenzyl)-4-oxo-4,5,6,7-tetrahydrothieno[3,2-c]pyridine-2-carboxylate* (**8b**). Following the same procedure used for the synthesis of **8a**, the reaction of 4-methoxy-1-(4-methoxybenzyl)-2-oxo-1,2,5,6-tetrahydropyridine-3-carbonitrile **7b** (354 mg, 1.30 mmol), NaOMe (5M in MeOH, 0.40 mL, 2.08 mmol), and methyl thioglycolate (0.21 mL, 2.34 mmol) gave the title compound **8b** (281 mg, 0.81 mmol, 62% yield) as a yellow oil: ^1^H-NMR (400 MHz, CDCl_3_) δ 7.24–7.22 (m, 2H), 6.88–6.86 (m, 2H), 4.62 (s, 2H), 3.82 (s, 3H), 3.80 (s, 3H), 3.50 (t, *J* = 6.9 Hz, 2H), 2.91 (t, *J* = 6.9 Hz, 2H); ^13^C-NMR (100 MHz, CDCl_3_) δ 164.52, 162.63, 159.13, 153.74, 150.99, 129.37, 129.13, 119.77, 114.11, 96.80, 55.28, 51.12, 48.38, 45.59, 24.95; LC/MS (ESI^+^): *m/z*: calcd for C_17_H_18_N_2_O_4_S: 346.40, [M+H]^+^; found: 347.05.

*8-Benzyl-3-phenyl-7,8-dihydropyrido[3',4':4,5]thieno[3,2-d]pyrimidine-4,9(3H,6H)-dione* (**9a**). To a pressure bottle containing methyl 3-amino-5-benzyl-4-oxo-4,5,6,7-tetrahydrothieno[3,2-c]pyridine-2-carboxylate **8a** (1g, 3.03 mmol), CH(OEt)_3_ (10 mL) was added, followed by aniline (0.524 mL, 5.75 mmol) and AcOH (1 mL). The reaction mixture was stirred and refluxed at 160 °C for 18 h. After the reaction, the mixture was evaporated then solidified with EtOAc and Et_2_O. The produced solid was filtered and dried *in vacuo* to give the title product **9a** (743 mg, 1.92 mmol, 63% yield) as a reddish solid: mp 172–173 °C; ^1^H-NMR (300 MHz, DMSO-*d_6_*) δ 8.44–7.51 (m, 5H), 7.39–7.26 (m, 5H), 4.71 (s, 2H), 3.62 (t, *J* = 6.6 Hz, 2H), 3.23 (t, *J* = 6.6 Hz, 2H); ^13^C-NMR (100 MHz, DMSO-*d_6_*) δ 159.61, 156.61, 156.48, 154.82, 149.69, 138.26, 137.32, 129.71, 129.49, 129.20, 128.08, 128.02, 127.63, 126.01, 122.13, 49.19, 46.26, 25.79; HRMS (ESI TOF-mass) calcd for C_22_H_17_N_3_O_2_S [M+Na]^+^ 410.0934, found 410.0936; purity (HPLC) 83.91%, *t*_R_ 18.00 min.

*8-Benzyl-3-(4-chlorophenyl)-7,8-dihydropyrido[3',4':4,5]thieno[3,2-d]pyrimidine-4,9(3H,6H)-dione* (**9b**). Following the same procedure used for the synthesis of **9a**, the reaction of methyl 3-amino-5-benzyl-4-oxo-4,5,6,7-tetrahydrothieno[3,2-c]pyridine-2-carboxylate **8a** (100 mg, 0.32 mmol), CH(OEt)_3_ (1 mL), 4-chloroaniline (72.7 mg, 0.57 mmol) and AcOH (0.1 mL) gave the title compound **9b** (89 mg, 0.21 mmol, 66% yield) as a yellowish solid: mp 220–223 °C; ^1^H-NMR (400 MHz, CDCl_3_) δ 8.27 (s, 1H), 7.54–7.27 (m, 9H), 4.80 (s, 2H), 3.63 (t, *J* = 6.7 Hz, 2H), 3.13 (t, *J* = 6.8 Hz, 2H); ^13^C-NMR (100 MHz, CDCl_3_) δ 160.06, 156.36, 155.24, 154.74, 148.31, 137.33, 135.55, 135.10, 129.93, 128.72, 128.46, 128.39, 127.65, 126.17, 122.65, 49.53, 45.64, 25.89; HRMS (ESI TOF-mass) calcd for C_22_H_16_ClN_3_O_2_S [M+Na]^+^ 444.0544, found 444.0545; purity (HPLC) 100.00%, *t*_R_ 19.21 min.

*8-Benzyl-3-(4-methoxyphenyl)-7,8-dihydropyrido[3',4':4,5]thieno[3,2-d]pyrimidine-4,9(3H,6H)-dione* (**9c**). Following the same procedure used for the synthesis of **9a**, the reaction of methyl 3-amino-5-benzyl-4-oxo-4,5,6,7-tetrahydrothieno[3,2-c]pyridine-2-carboxylate **8a** (100 mg, 0.32 mmol), CH(OEt)_3_ (1 mL), *p*-anisidine (72.7 mg, 0.57 mmol) and AcOH (0.1 mL) gave the title compound **9c** (44 mg, 0.11 mmol, 33% yield) as a reddish solid: mp 223–225 °C; ^1^H-NMR (400 MHz, CDCl_3_) δ 8.27 (s, 1H), 7.40–7.04 (m, 9H), 4.81 (s, 2H), 3.87 (s, 3H), 3.63 (t, *J* = 6.7 Hz, 2H), 3.14 (t, *J* = 6.7 Hz, 2H); ^13^C-NMR (100 MHz, CDCl_3_) δ 160.19, 160.17, 156.84, 154.92, 154.81, 149.05, 137.41, 129.35, 128.71, 128.39, 128.23, 127.61, 126.13, 122.79, 114.88, 55.64, 49.48, 45.64, 25.89; HRMS (ESI TOF-mass) calcd for C_23_H_19_N_3_O_3_S [M+Na]^+^ 440.1039, found 440.1039; purity (HPLC) 100.00%, *t*_R_ 18.09 min.

*8-Benzyl-3-(3-methoxyphenyl)-7,8-dihydropyrido[3',4':4,5]thieno[3,2-d]pyrimidine-4,9(3H,6H)-dione* (**9d**). Following the same procedure used for the synthesis of **9a**, the reaction of methyl 3-amino-5-benzyl-4-oxo-4,5,6,7-tetrahydrothieno[3,2-c]pyridine-2-carboxylate **8a** (200 mg, 0.63 mmol), CH(OEt)_3_ (2 mL), *m*-anisidine (135 μL, 1.21 mmol) and AcOH (0.2 mL) gave the title compound **9d** (53 mg, 0.13 mmol, 20% yield) as a white solid: mp 223–226 °C; ^1^H-NMR (400 MHz, CDCl_3_) δ 8.27 (s, 1H), 7.47–6.95 (m, 9H), 4.81 (s, 2H), 3.85 (s, 3H), 3.63 (t, *J* = 6.8 Hz, 2H), 3.14 (t, *J* = 6.7 Hz, 2H); ^13^C-NMR (100 MHz, CDCl_3_) δ 160.43, 160.17, 156.50, 154.98, 154.78, 148.72, 137.72, 137.38, 130.45, 128.72, 128.39, 127.63, 126.18, 122.86, 119.15, 115.39, 112.93, 55.60, 49.49, 45.63, 25.91; HRMS (ESI TOF-mass) calcd for C_23_H_19_N_3_O_3_S [M+Na]^+^ 440.1039, found 440.1038; purity (HPLC) 99.64%, *t*_R_ 18.31 min.

*8-(4-Methoxybenzyl)-3-phenyl-7,8-dihydropyrido[3',4':4,5]thieno[3,2-d]pyrimidine-4,9(3H,6H)-dione* (**9e**). Following the same procedure used for the synthesis of **9a**, the reaction of methyl 3-amino-5-(4-methoxybenzyl)-4-oxo-4,5,6,7-tetrahydrothieno[3,2-c]pyridine-2-carboxylate **8b** (118 mg, 0.34 mmol), CH(OEt)_3_ (1.2 mL), aniline (62 μL, 0.68 mmol) and AcOH (0.12 mL) gave the title compound **9e** (95 mg, 0.23 mmol, 67% yield) as a yellowish solid: mp 227–230 °C; ^1^H-NMR (400 MHz, DMSO-*d_6_*) δ 8.27 (s, 1H), 7.58–6.85 (m, 9H), 4.73 (s, 2H), 3.79 (s, 3H), 3.61 (t, *J* = 6.7 Hz, 2H), 3.12 (t, *J* = 6.8 Hz, 2H); ^13^C-NMR (100 MHz, CDCl_3_) δ 160.16, 159.18, 156.56, 154.96, 154.78, 148.80, 136.70, 134.10, 129.76, 129.70, 129.43, 127.06, 126.22, 122.87, 114.09, 55.31, 48.87, 45.43, 25.91; HRMS (ESI TOF-mass) calcd for C_23_H_19_N_3_O_3_S [M+Na]^+^ 440.1039, found 440.1039; purity (HPLC) 98.75%, *t*_R_ 17.88 min.

*3-(4-Chlorophenyl)-8-(4-methoxybenzyl)-7,8-dihydropyrido[3',4':4,5]thieno[3,2-d]pyrimidine-4,9(3H,6H)-dione* (**9f**). Following the same procedure used for the synthesis of **9a**, the reaction of methyl 3-amino-5-(4-methoxybenzyl)-4-oxo-4,5,6,7-tetrahydrothieno[3,2-c]pyridine-2-carboxylate **8b** (100 mg, 0.29 mmol), CH(OEt)_3_ (1 mL), 4-chloroaniline (74 mg, 0.58 mmol) and AcOH (0.1 mL) gave the title compound **9f** (68 mg, 0.15 mmol, 52% yield) as a yellowish solid: mp 269–272 °C; ^1^H-NMR (400 MHz, CDCl_3_) δ 8.24 (s, 1H), 7.55–6.86 (m, 8H), 4.74 (s, 2H), 3.80 (s, 3H), 3.62 (t, *J* = 6.7 Hz, 2H), 3.13 (t, *J* = 6.7 Hz, 2H); ^13^C-NMR (100 MHz, CDCl_3_) δ 159.98, 159.17, 156.36, 155.19, 154.72, 148.27, 135.53, 135.11, 129.91, 129.77, 129.42, 128.47, 126.24, 122.61, 114.08, 55.31, 48.92, 45.46, 25.88; HRMS (ESI TOF-mass) calcd for C_23_H_18_ClN_3_O_3_S [M+Na]^+^ 474.0650, found 474.0652; purity (HPLC) 100.00%, *t*_R_ 19.03 min.

*8-(4-Methoxybenzyl)-3-(4-methoxyphenyl)-7,8-dihydropyrido[3',4':4,5]thieno[3,2-d]pyrimidine-4,9(3H,6H)-dione* (**9g**). Following the same procedure used for the synthesis of **9a**, the reaction of methyl 3-amino-5-(4-methoxybenzyl)-4-oxo-4,5,6,7-tetrahydrothieno[3,2-c]pyridine-2-carboxylate **8b** (100 mg, 0.29 mmol), CH(OEt)_3_ (1 mL), p-anisidine (73 mg, 0.58 mmol) and AcOH (0.1 mL) gave the title compound **9g** (67 mg, 0.15 mmol, 51% yield) as a reddish solid: mp 225–228 °C; ^1^H-NMR (400 MHz, CDCl_3_) δ 8.25 (s, 1H), 7.33–7.30 (m, 4H), 7.06–6.84 (m, 4H), 4.73 (s, 2H), 3.86 (s, 3H), 3.78 (s, 3H), 3.60 (t, *J* = 6.7 Hz, 2H), 3.12 (t, *J* = 6.7 Hz, 2H); ^13^C-NMR (100 MHz, CDCl_3_) δ 160.18, 160.16, 159.17, 156.85, 154.83, 154.82, 149.04, 129.76, 129.47, 129.35, 128.20, 126.23, 122.82, 114.89, 114.08, 55.63, 55.31, 48.86, 45.44, 25.91; HRMS (ESI TOF-mass) calcd for C_24_H_21_N_3_O_4_S [M+Na]^+^ 470.1145, found 470.1146; purity (HPLC) 100.00%, *t*_R_ 17.95 min.

*8-(4-Methoxybenzyl)-3-(3-methoxyphenyl)-7,8-dihydropyrido[3',4':4,5]thieno[3,2-d]pyrimidine-4,9(3H,6H)-dione* (**9h**). Following the same procedure used for the synthesis of **9a**, the reaction of methyl 3-amino-5-(4-methoxybenzyl)-4-oxo-4,5,6,7-tetrahydrothieno[3,2-c]pyridine-2-carboxylate **8b** (100 mg, 0.29 mmol), CH(OEt)_3_ (1 mL), m-anisidine (65 μL, 0.58 mmol) and AcOH (0.1 mL) gave the title compound **9h** (66 mg, 0.15 mmol, 51% yield) as a yellowish solid: mp 171–175 °C; ^1^H-NMR (400 MHz, CDCl_3_) δ 8.28 (s, 1H), 7.46–6.84 (m, 8H), 4.74 (s, 2H), 3.85 (s, 3H), 3.79 (s, 3H), 3.61 (t, *J* = 6.7 Hz, 2H), 3.12 (t, *J* = 6.8 Hz, 2H); ^13^C-NMR (100 MHz, CDCl_3_) δ 160.42, 160.12, 159.16, 156.51, 154.98, 154.72, 148.77, 137.71, 130.44, 129.76, 129.44, 126.20, 122.81, 119.15, 115.41, 114.08, 112.92, 55.60, 55.31, 48.87, 45.44, 25.89; HRMS (ESI TOF-mass) calcd for C_24_H_21_N_3_O_4_S [M+Na]^+^ 470.1145, found 470.1146; purity (HPLC) 98.23%, *t*_R_ 18.13 min.

### 3.2. mGluR1 Assay using FDSS6000

Chem-3 cells which stably express mGluR1 were purchased from Milipore Co. (Billerica, MA, USA). Cells were grown in DMEM medium supplemented with 10% (v/v) fetal bovine serum, penicillin (100 U/mL), streptomycin (100 μg/mL), and puromycin (10 μg/mL) at 37 °C in a humid atmosphere of 5% CO_2_ and 95% air. For calcium assay, cells were harvested and dispensed into 96-well black wall clear bottom plates at a density of 40,000 cells per a well. After 18 h of incubation, cells were treated with Calcium-5 assay reagent, which is prepared by manufacture’s instruction (Molecular Devices Co. Sunnyvale, CA, USA). During fluorescence-based FDSS6000 assay, mGluR1 was activated using a high concentration of l-glutamate (10 μM) in HBSS, and proper concentrations of synthesized compounds were treated to cells 75 s before mGluR activation. All data were collected and analyzed using FDSS6000 and related software (Hamamatsu Photonics, Hamamatsu, Japan).

## 4. Conclusions

In this proof-of-concept study, a synthetic protocol was devised to allow access to a fused tricyclic heterocycle composed of dihydropyridone, thiophene and pyrimidinone. Also, even though only a small set of the dihydropyridothienopyrimidin-4,9-dione derivatives were synthesized, promising biological activity was identified in the title compounds. Thus, by using variously substituted benzylamines and anilines, an extensive structure-activity relationship study is warranted to investigate the pharmacological versatility of the dihydropyridothienopyrimidin-4,9-dione derivatives.

## Supplementary Materials

Supplementary materials can be accessed at: http://www.mdpi.com/1420-3049/20/03/5074/s1.
