# Supplementary material for: Synthesis of Novel Dihydropyridothienopyrimidin-4,9-dione Derivatives
Source: Molecules. 2015 Mar 19;20(3):5074–84. doi: 10.3390/molecules20035074 (PMC6272423; doi:10.3390/molecules20035074)
Supplement: Supplementary file 1 [file molecules-20-05074-s001.pdf]

Supporting Information

^{1}H- and ^{13}C-NMR spectra of compounds **4a** and **4b**……………..………..…………….**S1–S2**
^{1}H- and ^{13}C-NMR spectra of compounds **5a** and **5b**…………………...………………..**S3–S4**
^{1}H- and ^{13}C-NMR spectra of compounds **6a** and **6b**……………………………………….**S5–S6**
^{1}H- and ^{13}C-NMR spectra of compounds **7a** and **7b**……………………………………….**S7–S8**
^{1}H- and ^{13}C-NMR spectra of compounds **8a** and **8b**……………..……..…… ………...**S9–S10**
^{1}H- and ^{13}C-NMR spectra of compounds **9a~9h**……………..………..……………………**S11–S18**

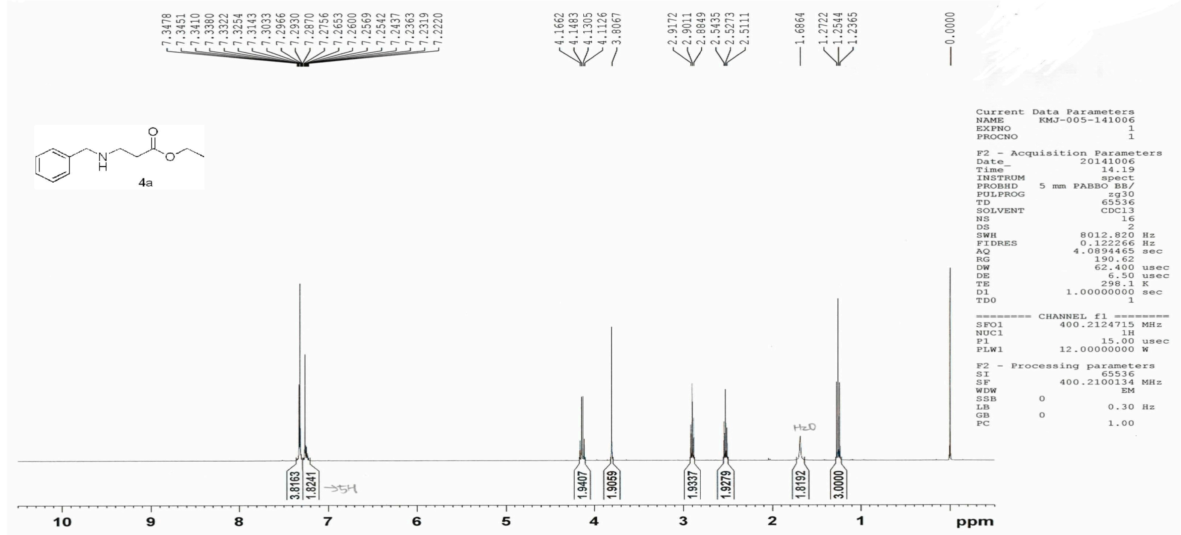

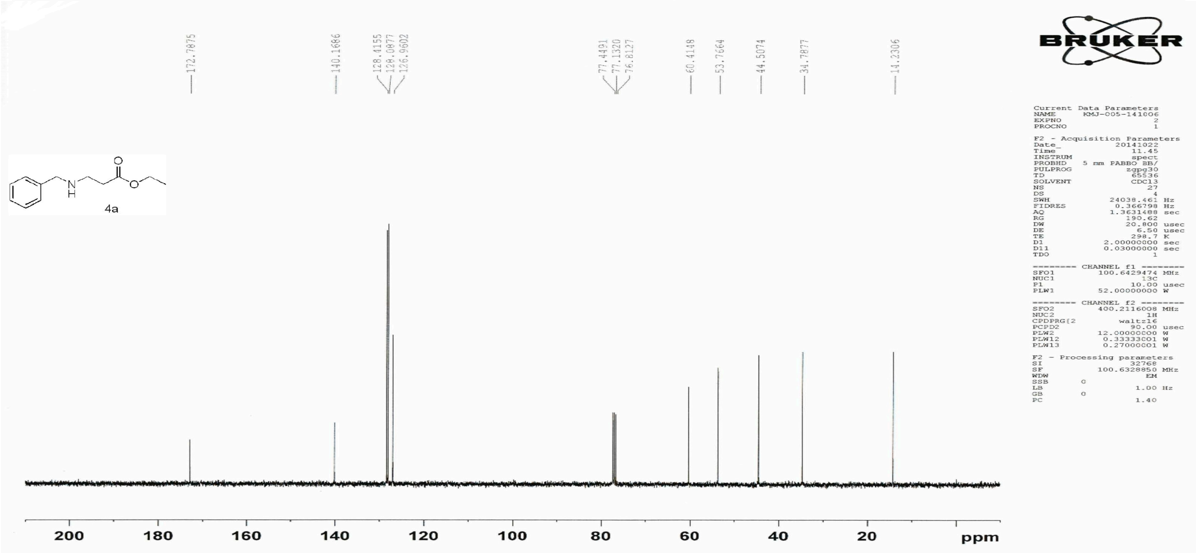

Figure S1. ^{1}H- (upper) and ^{13}C-NMR spectra (lower) of compounds **4a**.

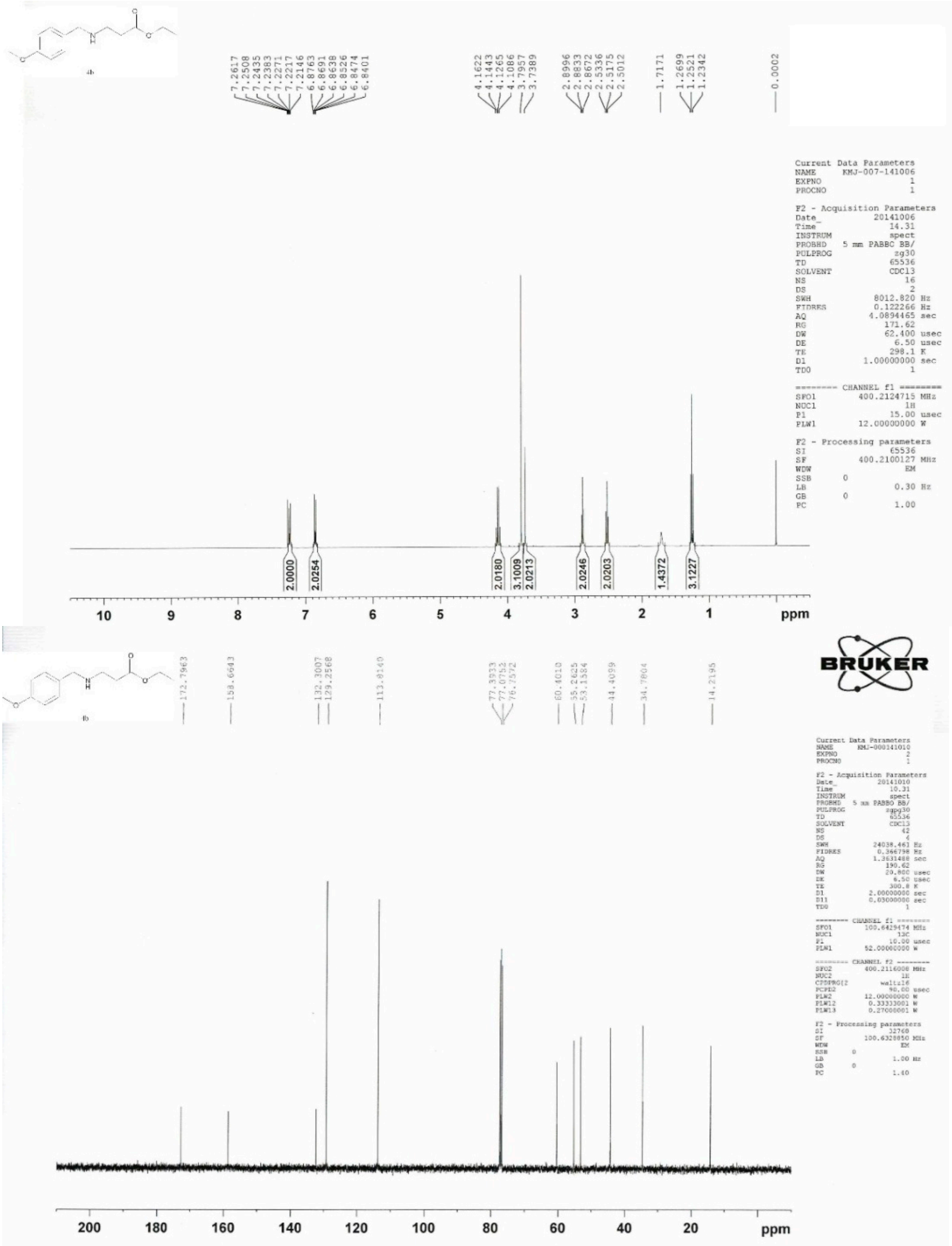

Figure S2. ^{1}H- (upper) and ^{13}C-NMR spectra (lower) of compounds **4b**.

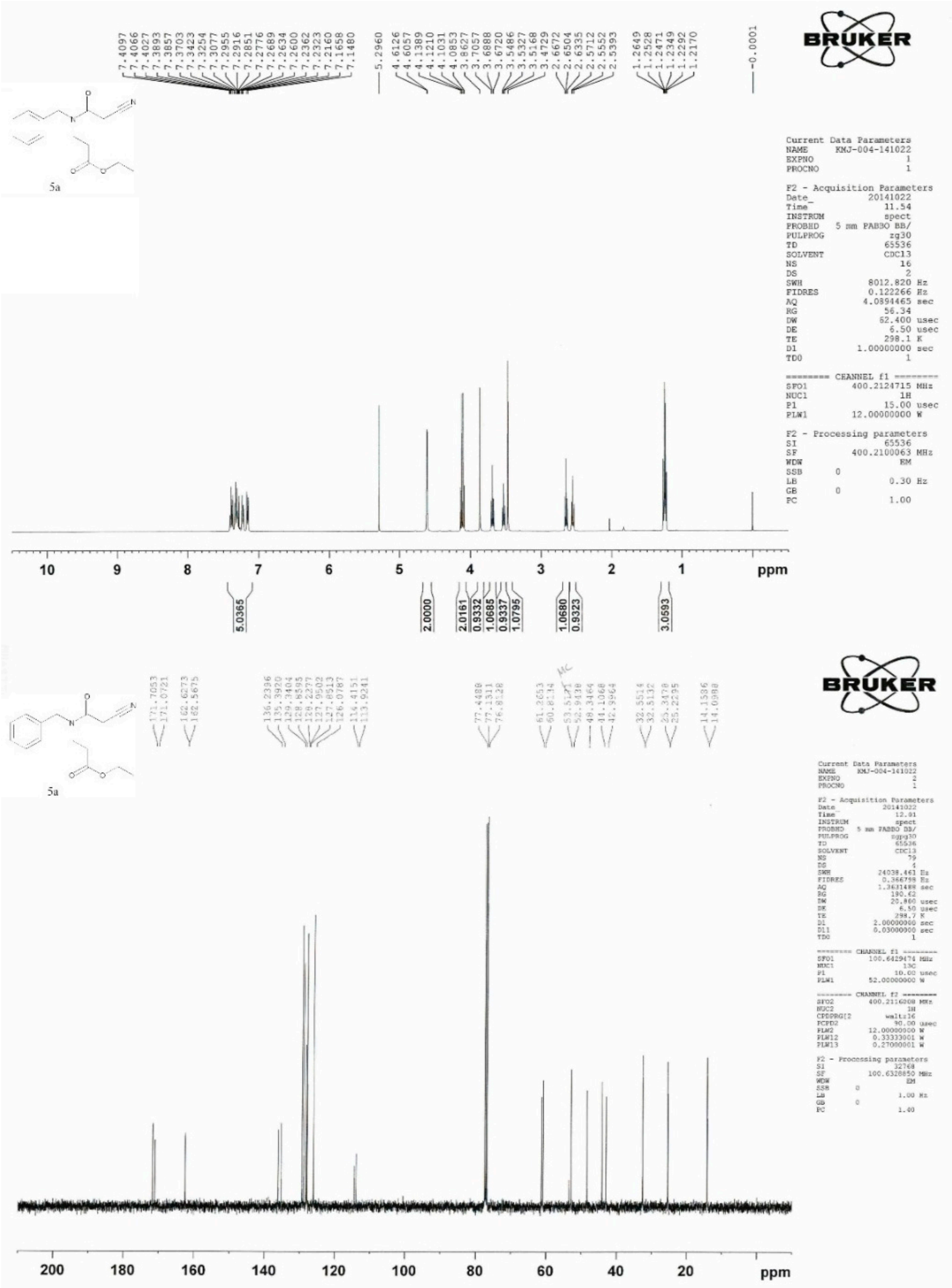

Figure S3. ^{1}H- (upper) and ^{13}C-NMR spectra (lower) of compounds **5a**.

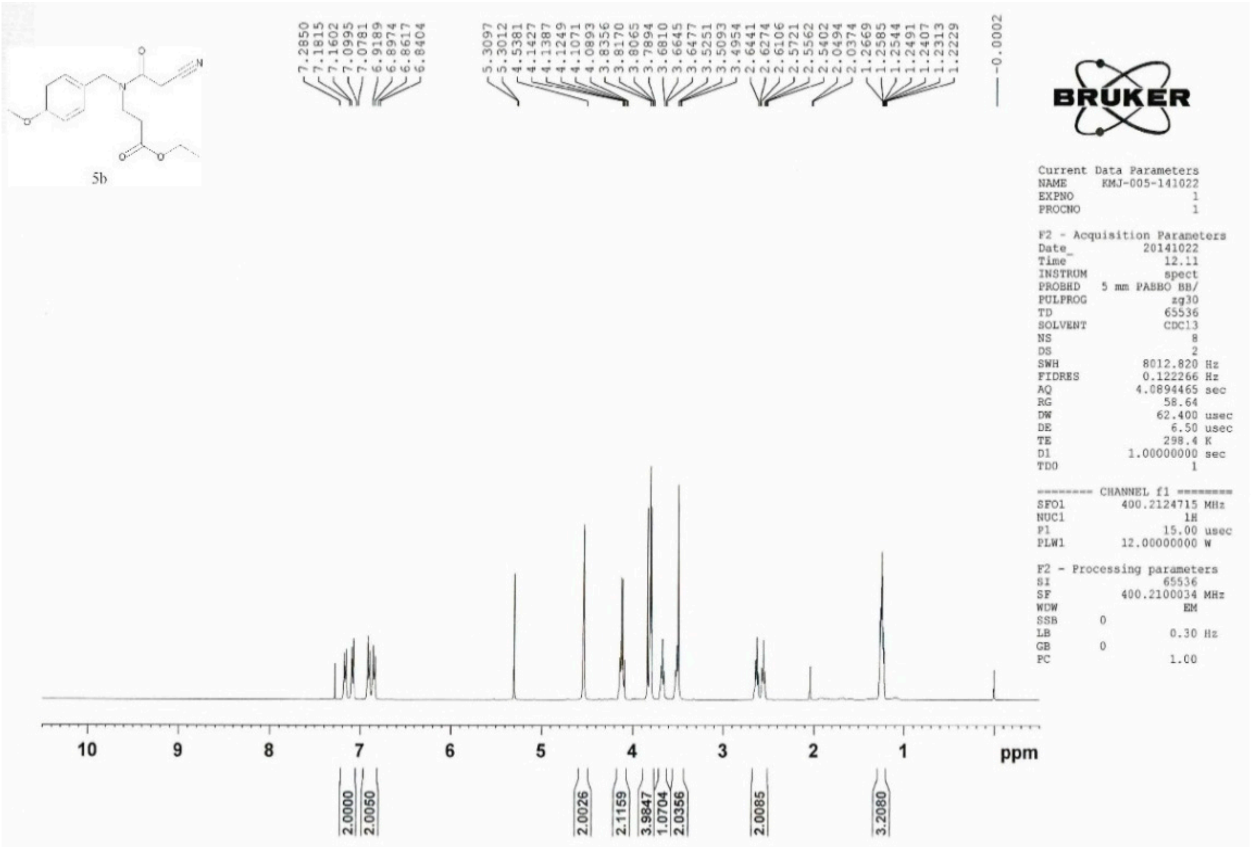

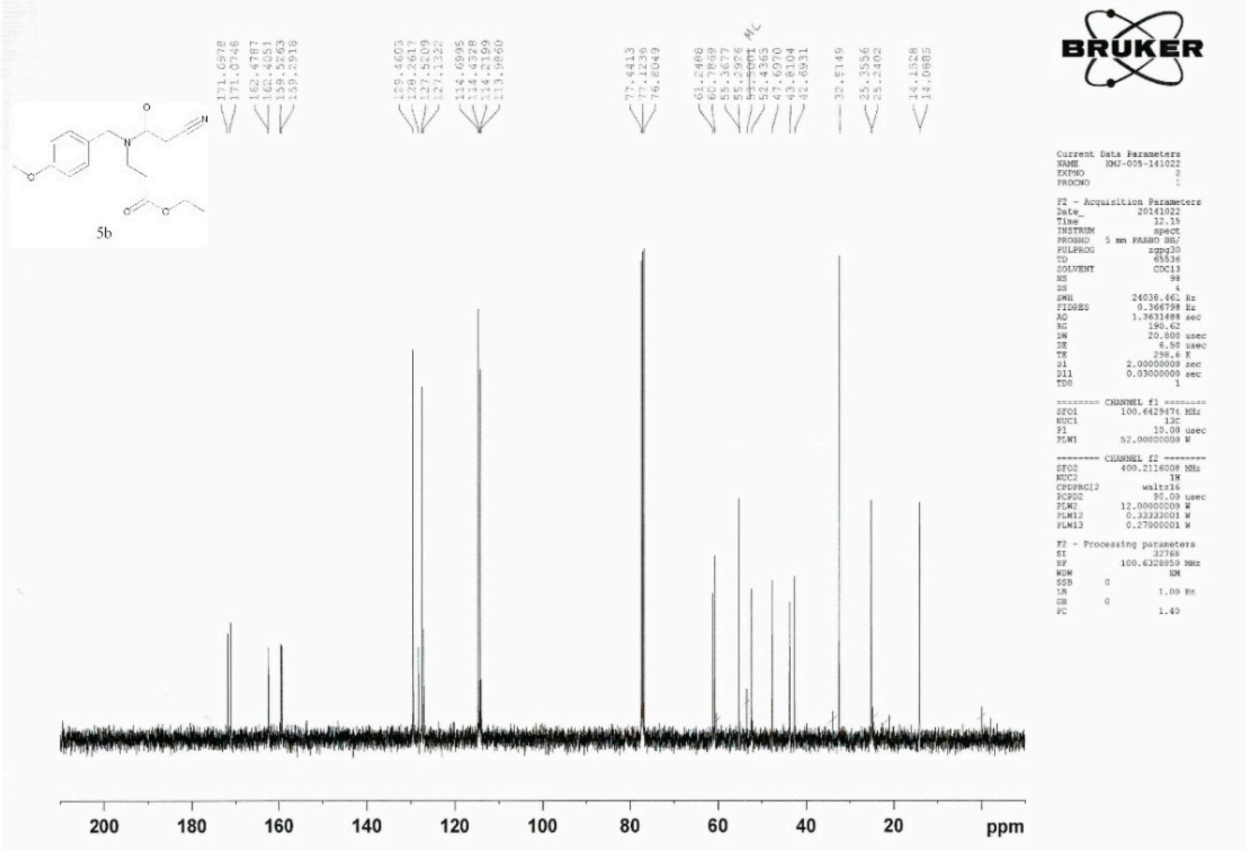

Figure S4. ^{1}H- (upper) and ^{13}C-NMR spectra (lower) of compounds **5b**.

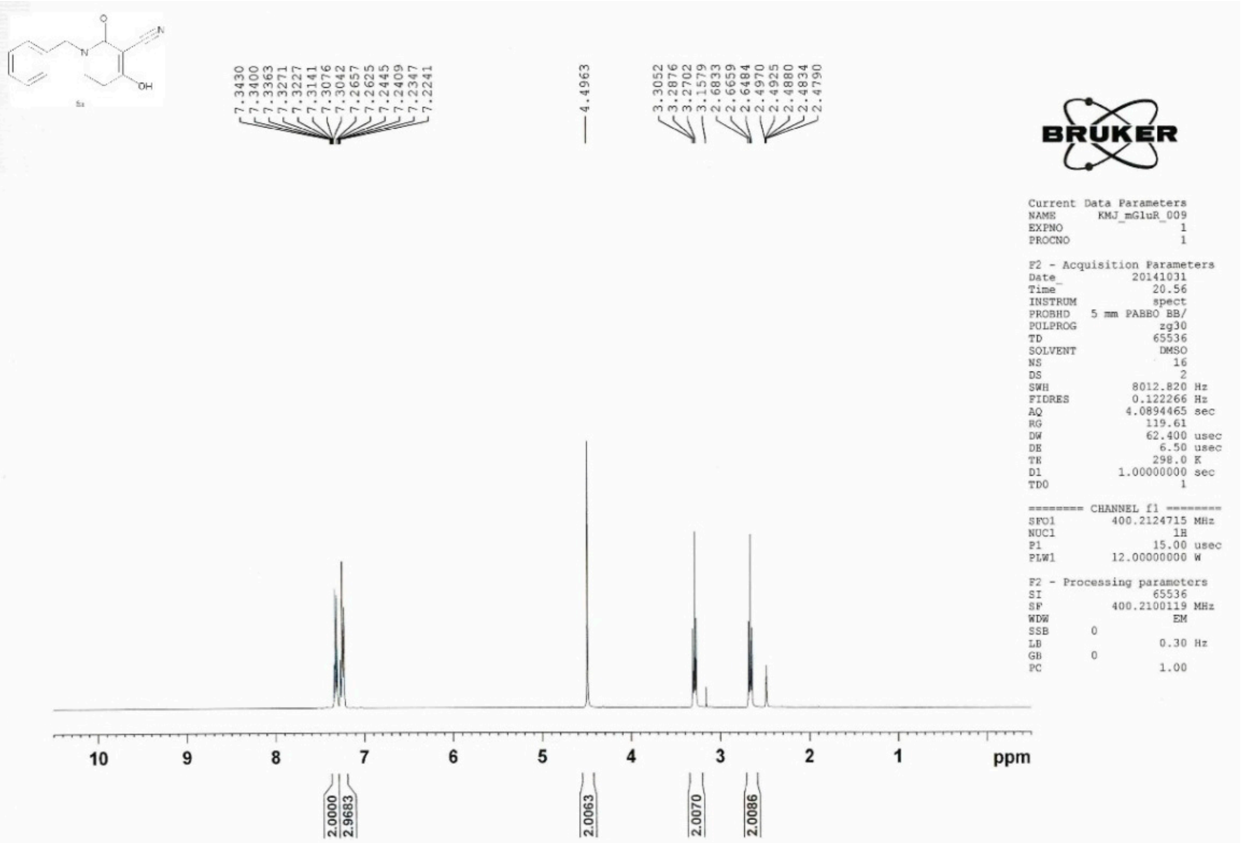

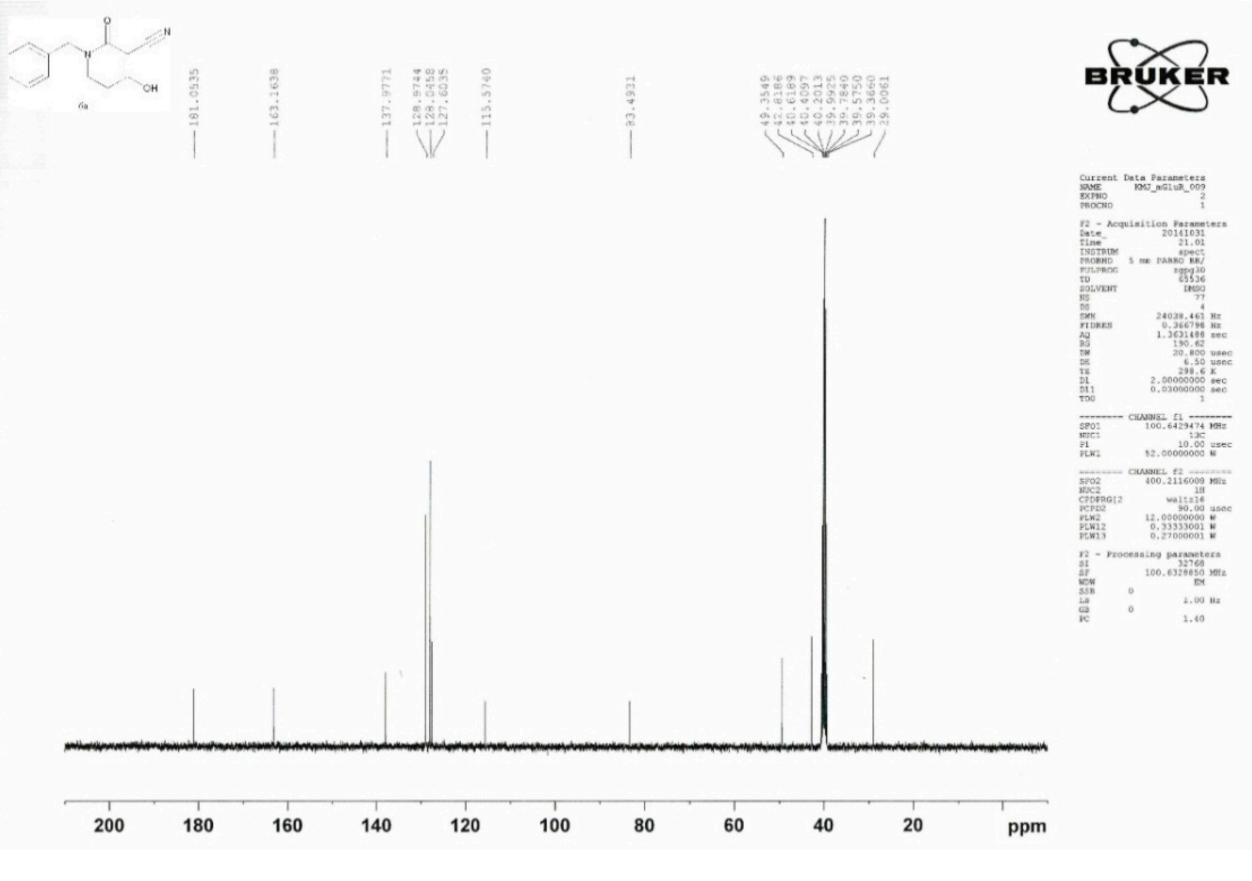

Figure S5. ^{1}H- (upper) and ^{13}C-NMR spectra (lower) of compounds **6a**.

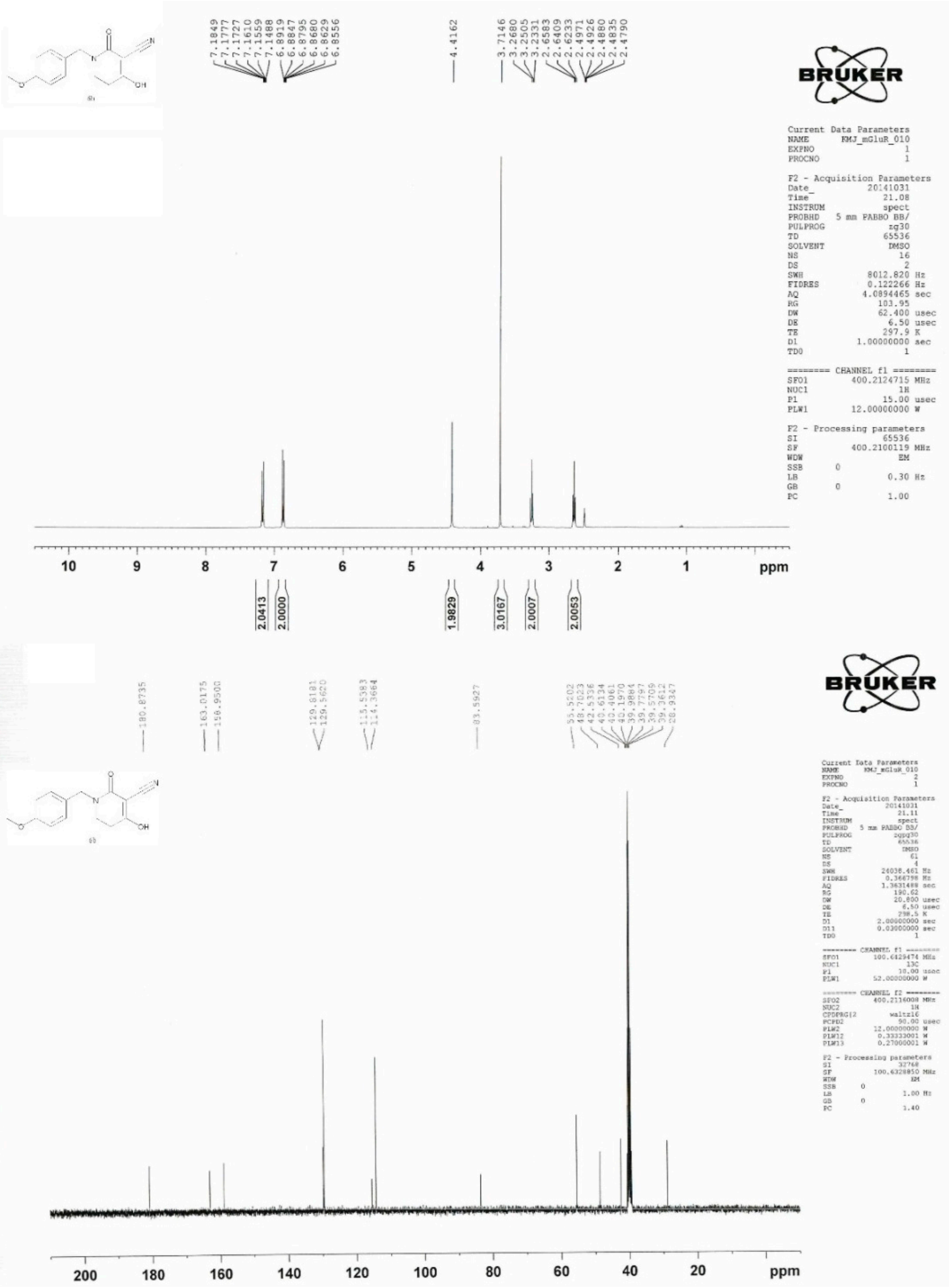

Figure S6. ^{1}H- (upper) and ^{13}C-NMR spectra (lower) of compounds **6b**.

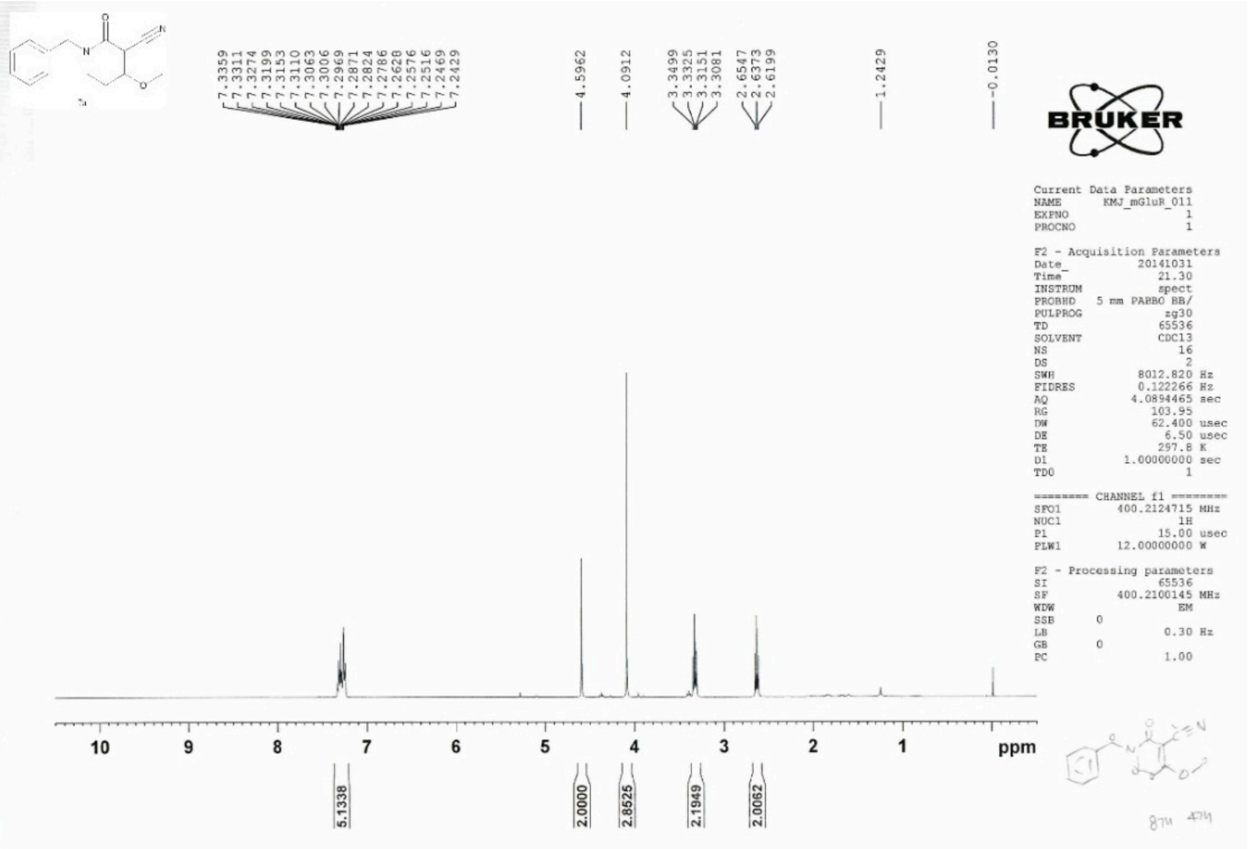

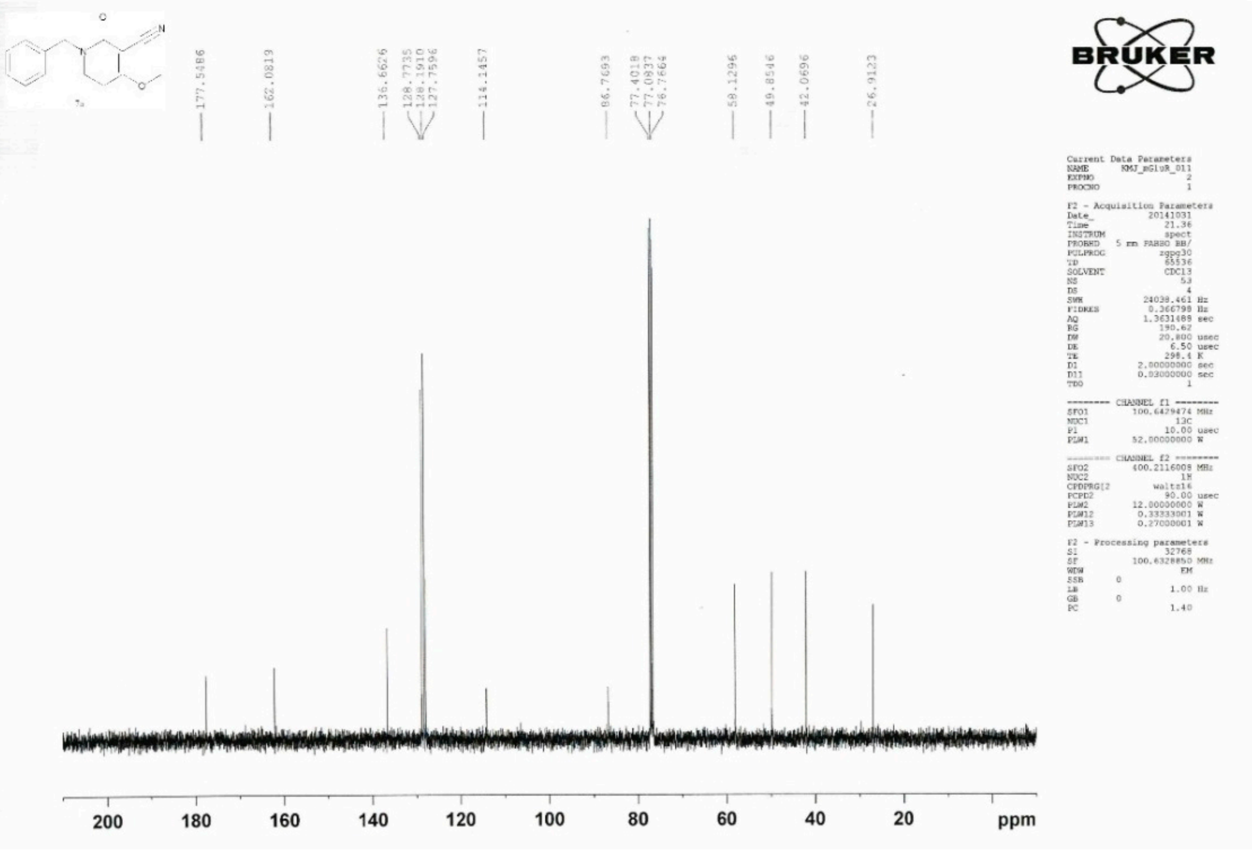

Figure S7. ^{1}H- (upper) and ^{13}C-NMR spectra (lower) of compounds **7a**.

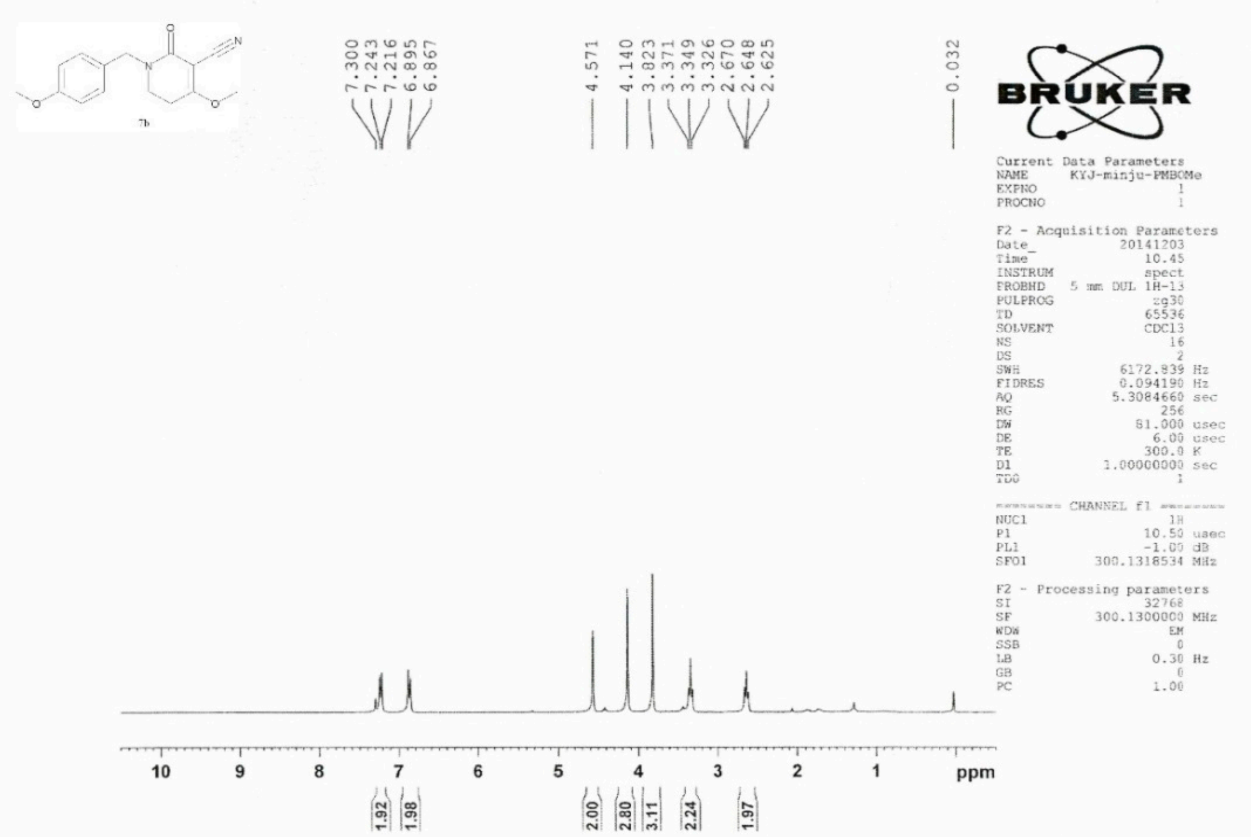

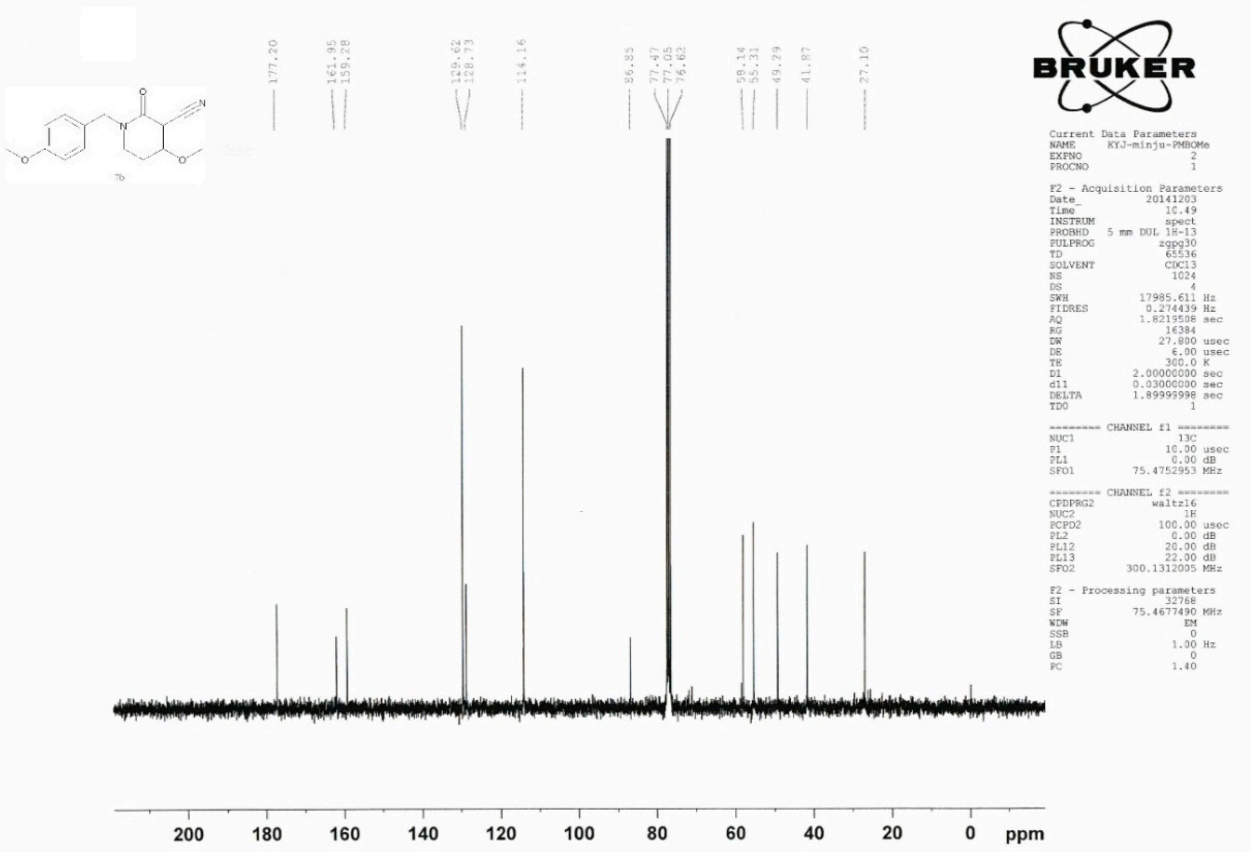

Figure S8. ^{1}H- (upper) and ^{13}C-NMR spectra (lower) of compounds **7b**.

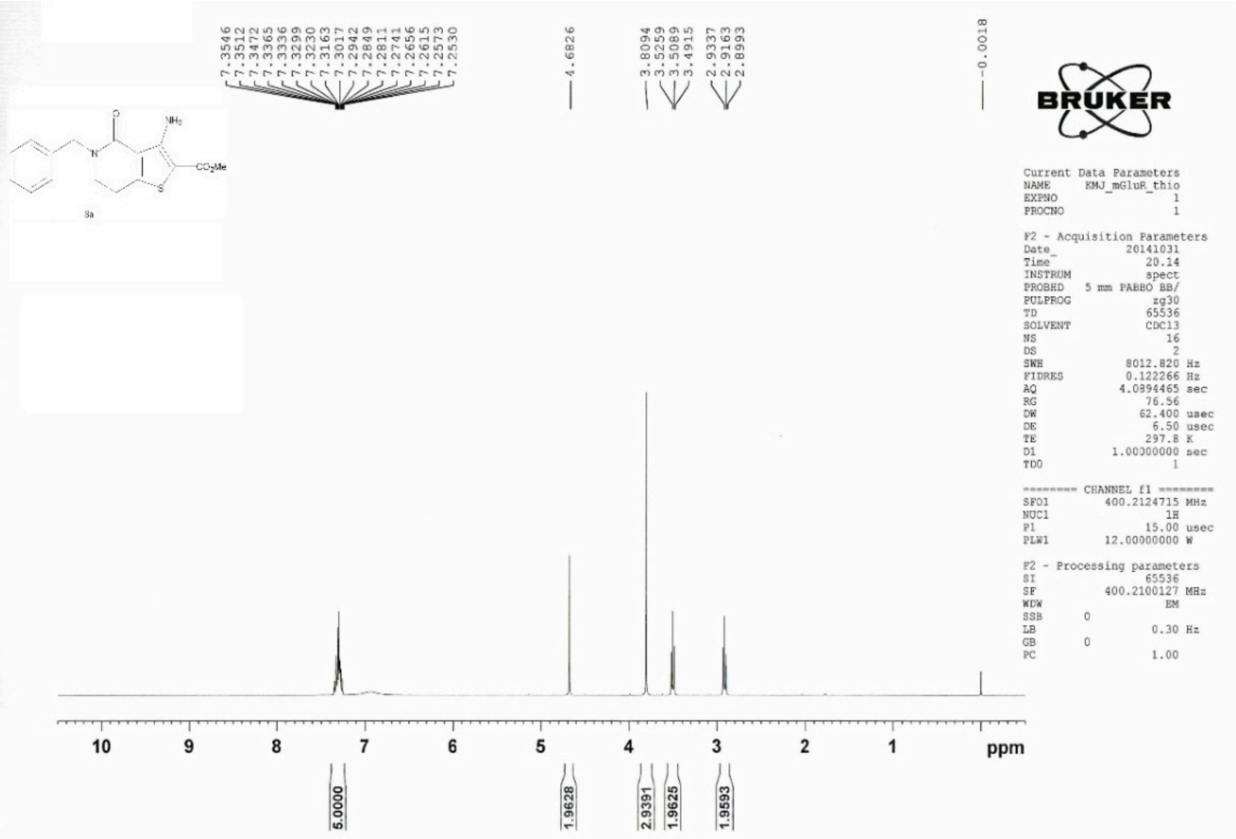

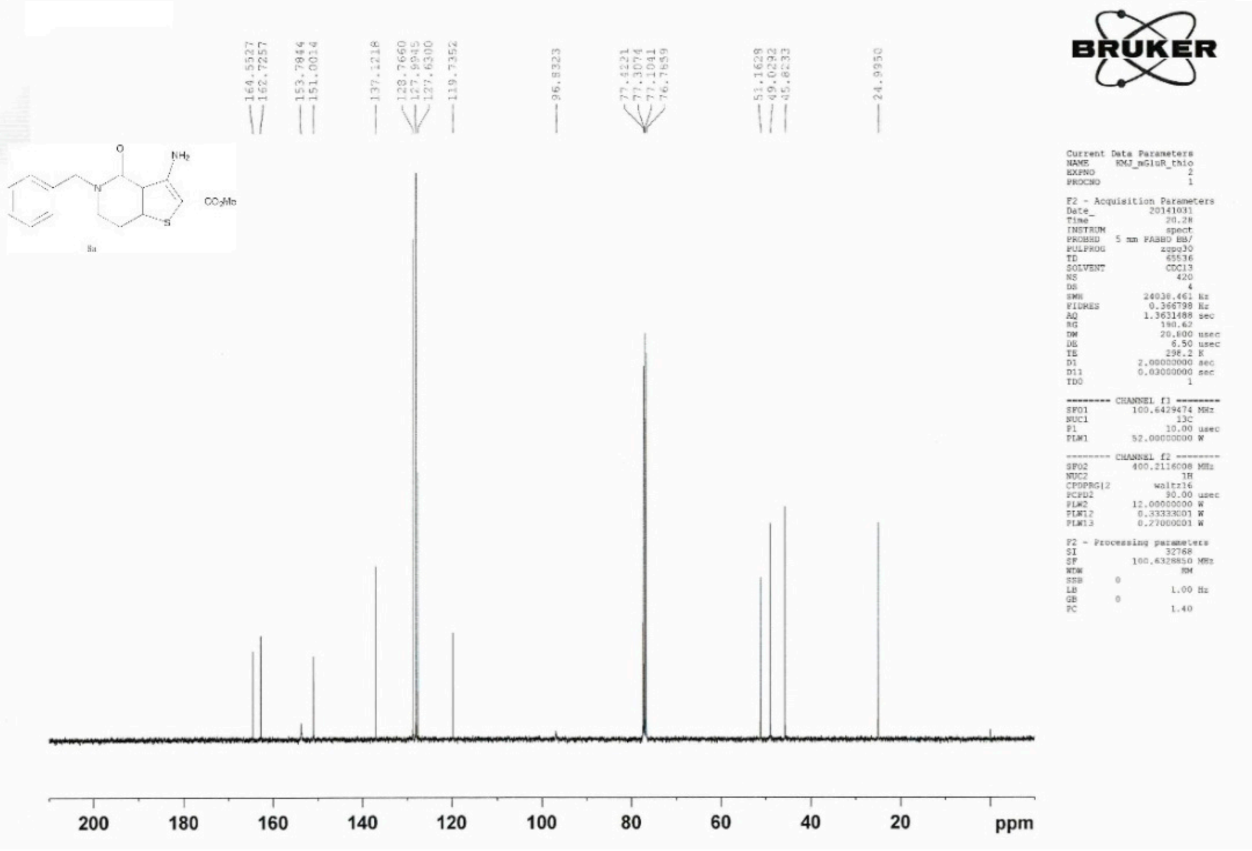

Figure S9. ^{1}H- (upper) and ^{13}C-NMR spectra (lower) of compounds **8a**.

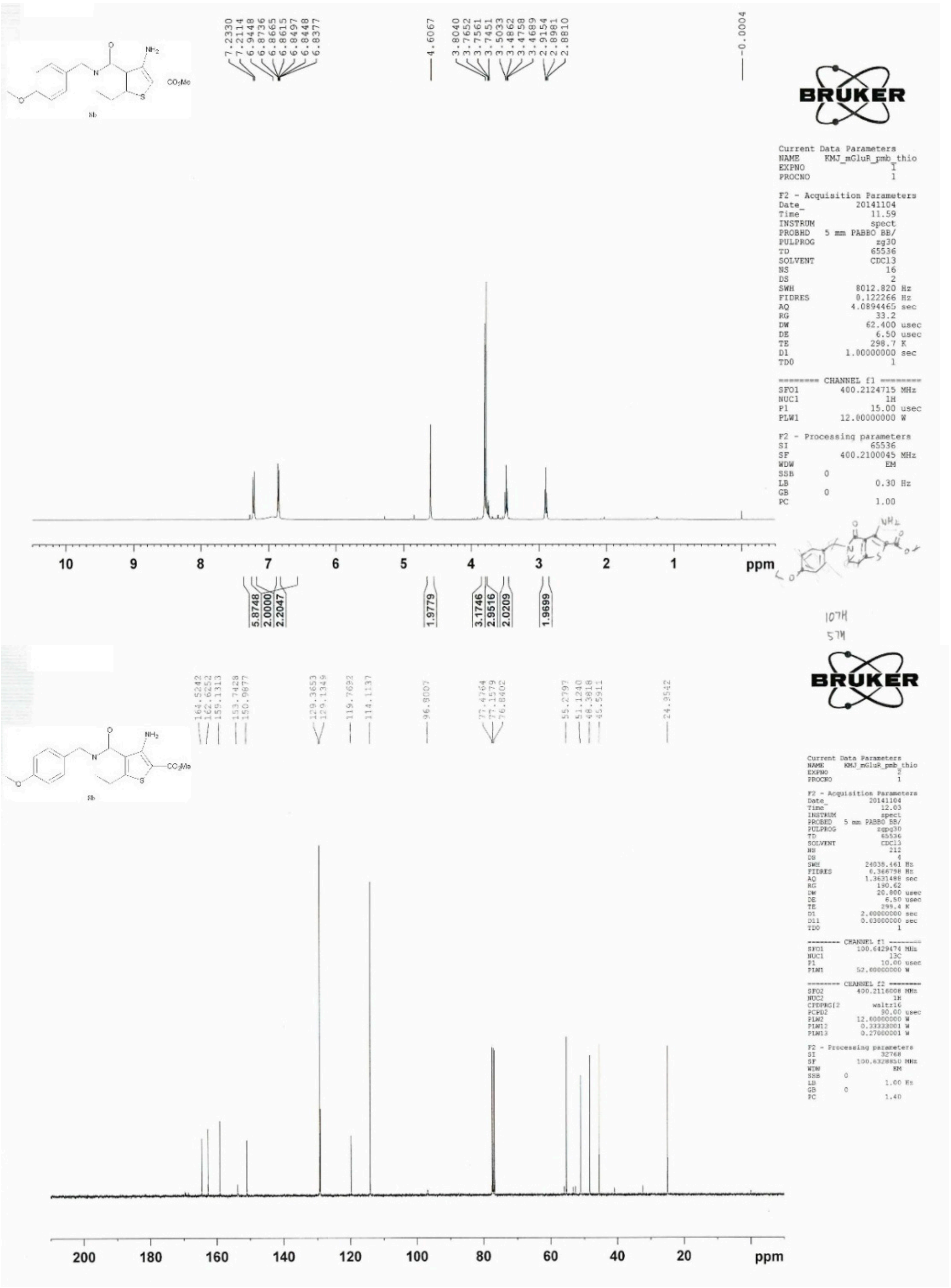

Figure S10. ^{1}H- (upper) and ^{13}C-NMR spectra (lower) of compounds **8b**.

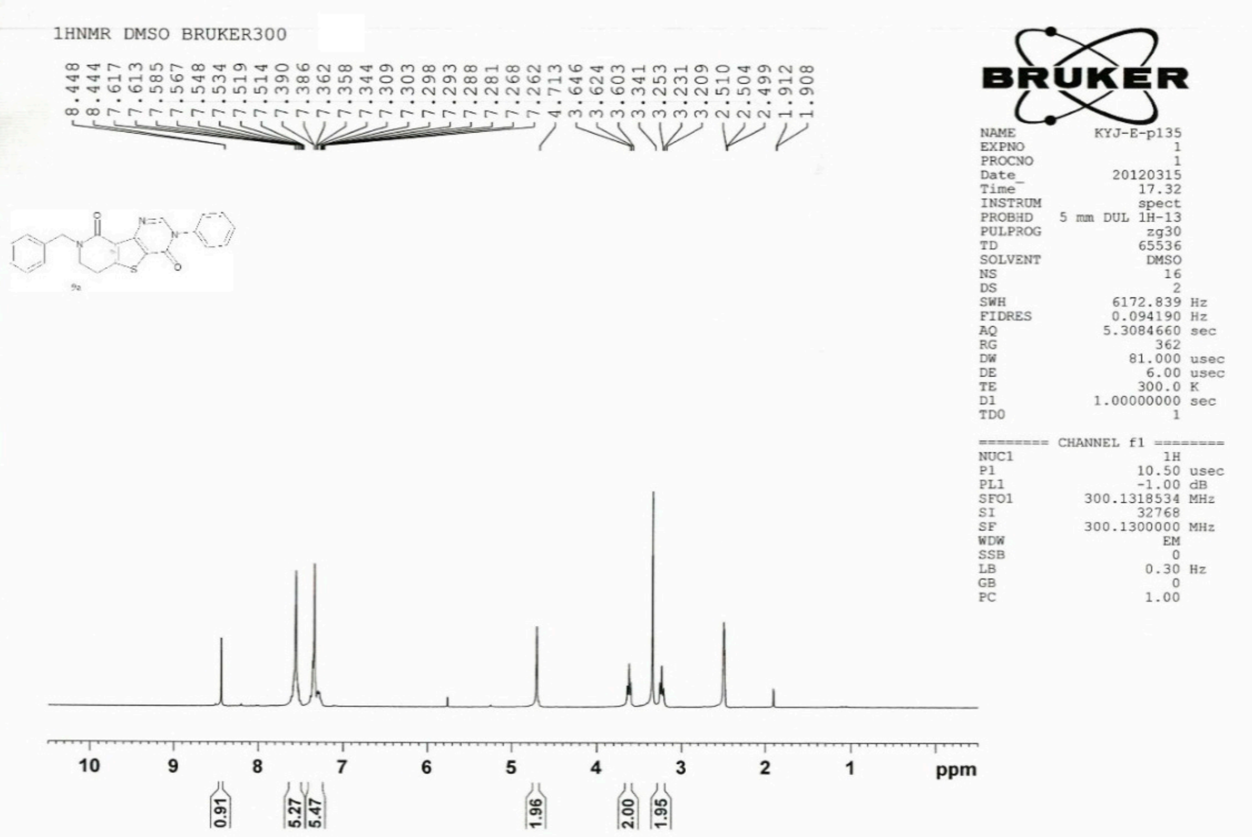

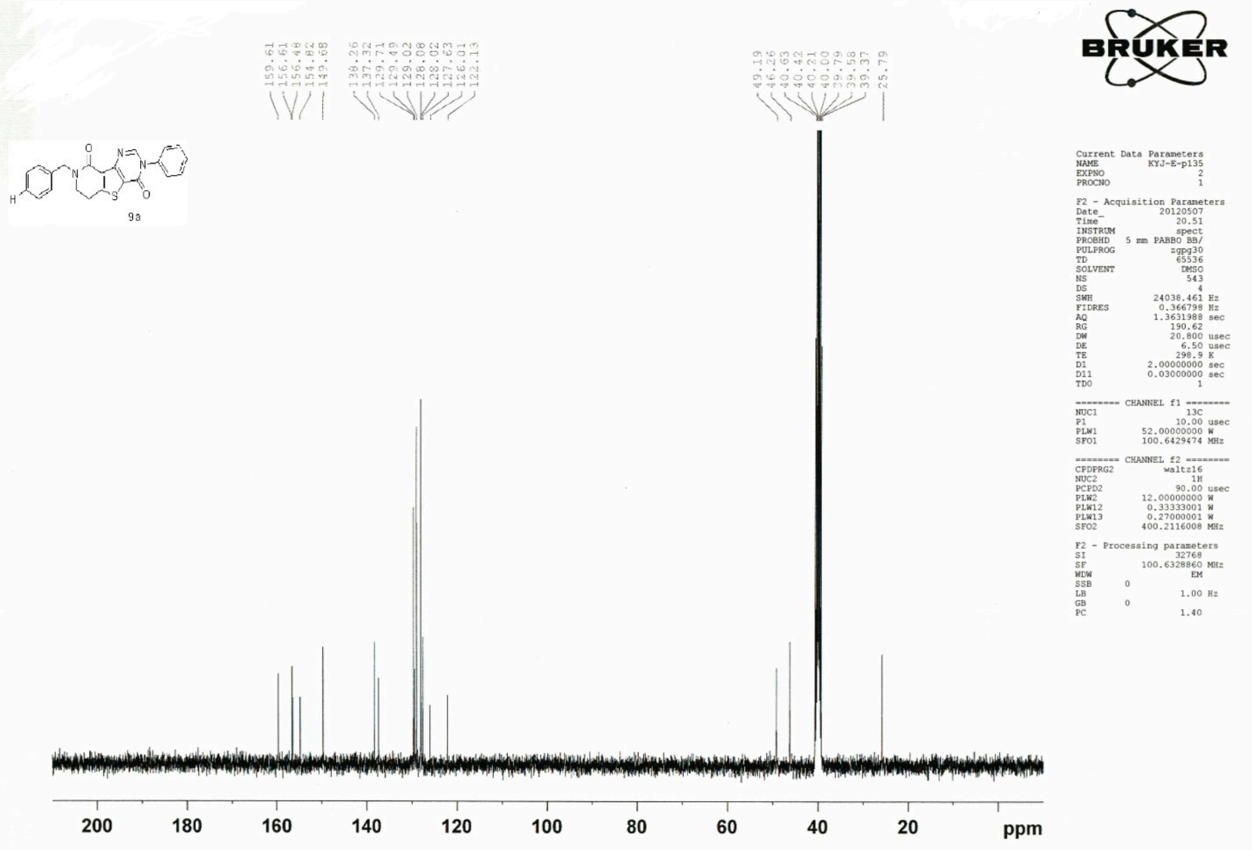

Figure S11. ^{1}H- (upper) and ^{13}C-NMR spectra (lower) of compounds **9a**.

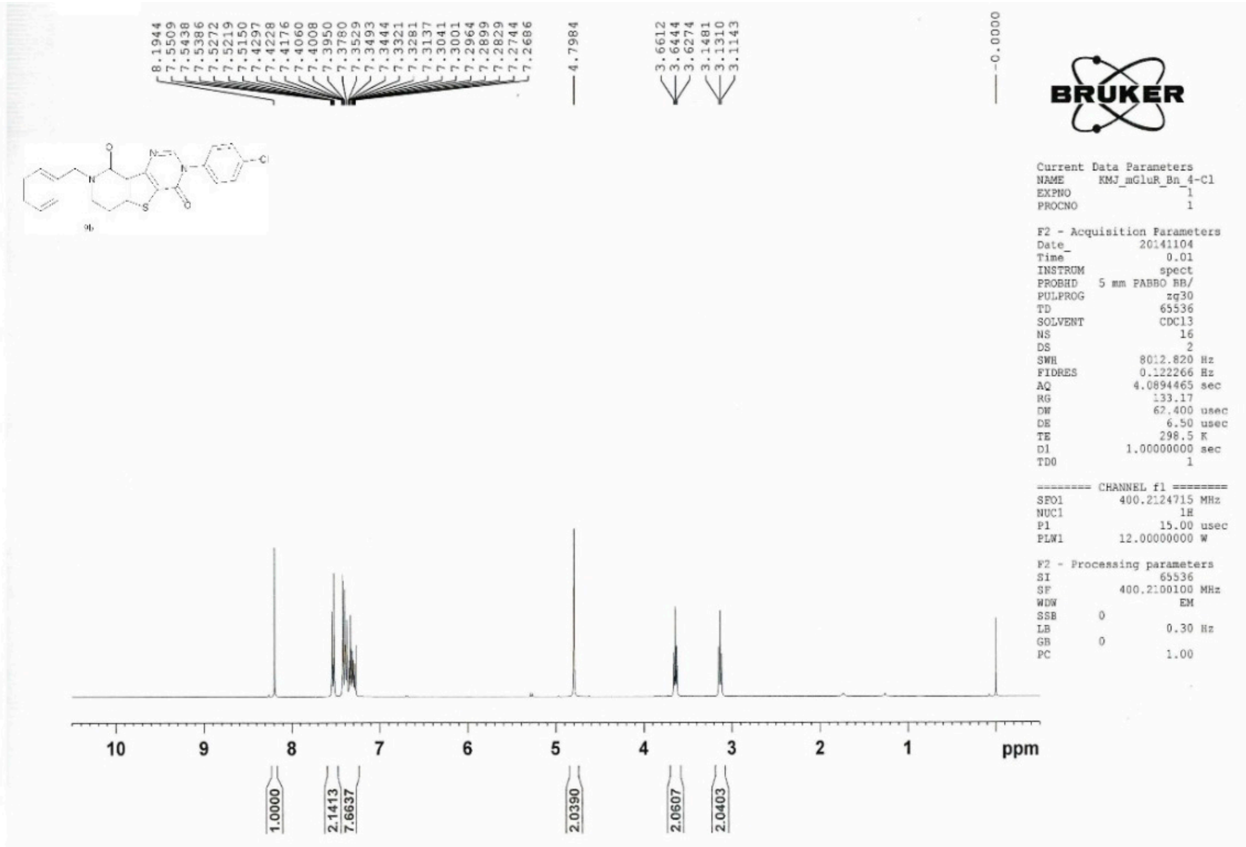

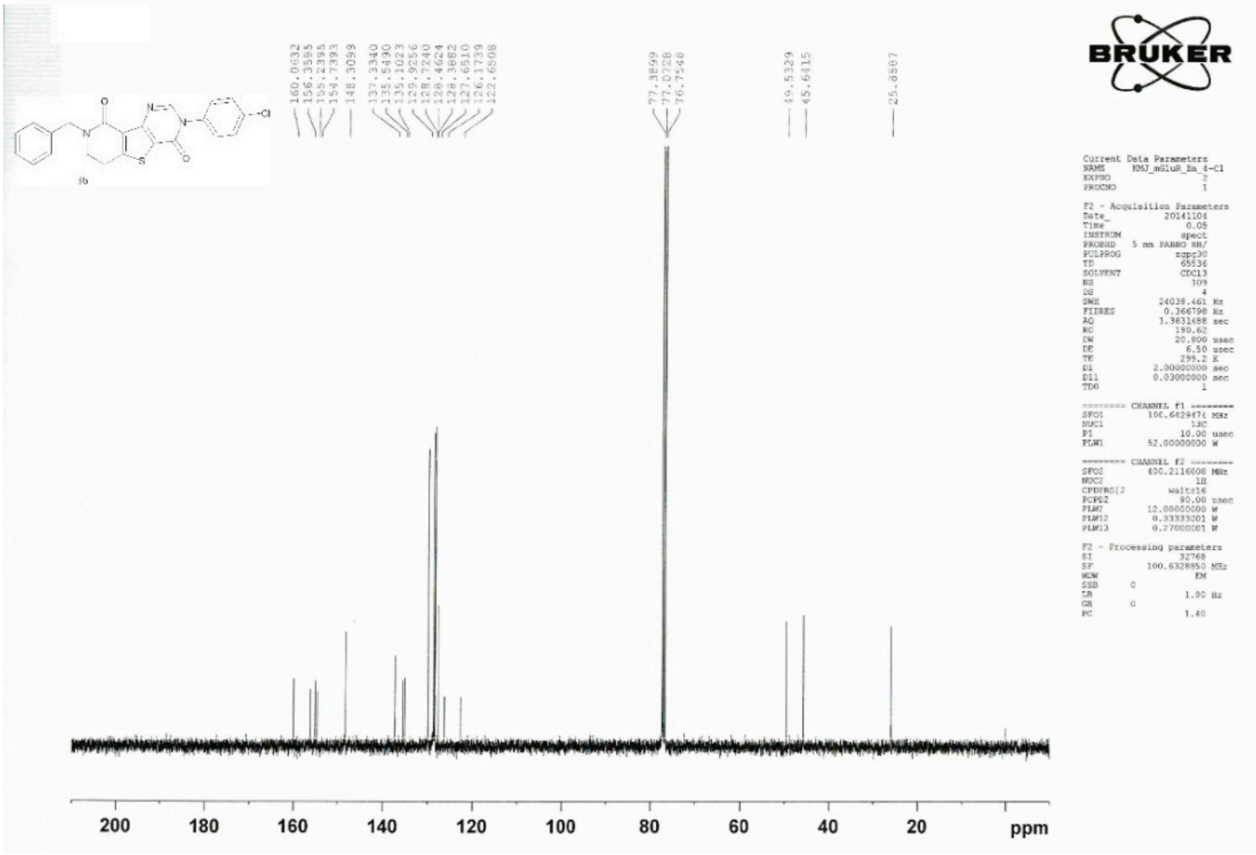

Figure S12. ^{1}H- (upper) and ^{13}C-NMR spectra (lower) of compounds **9b**.

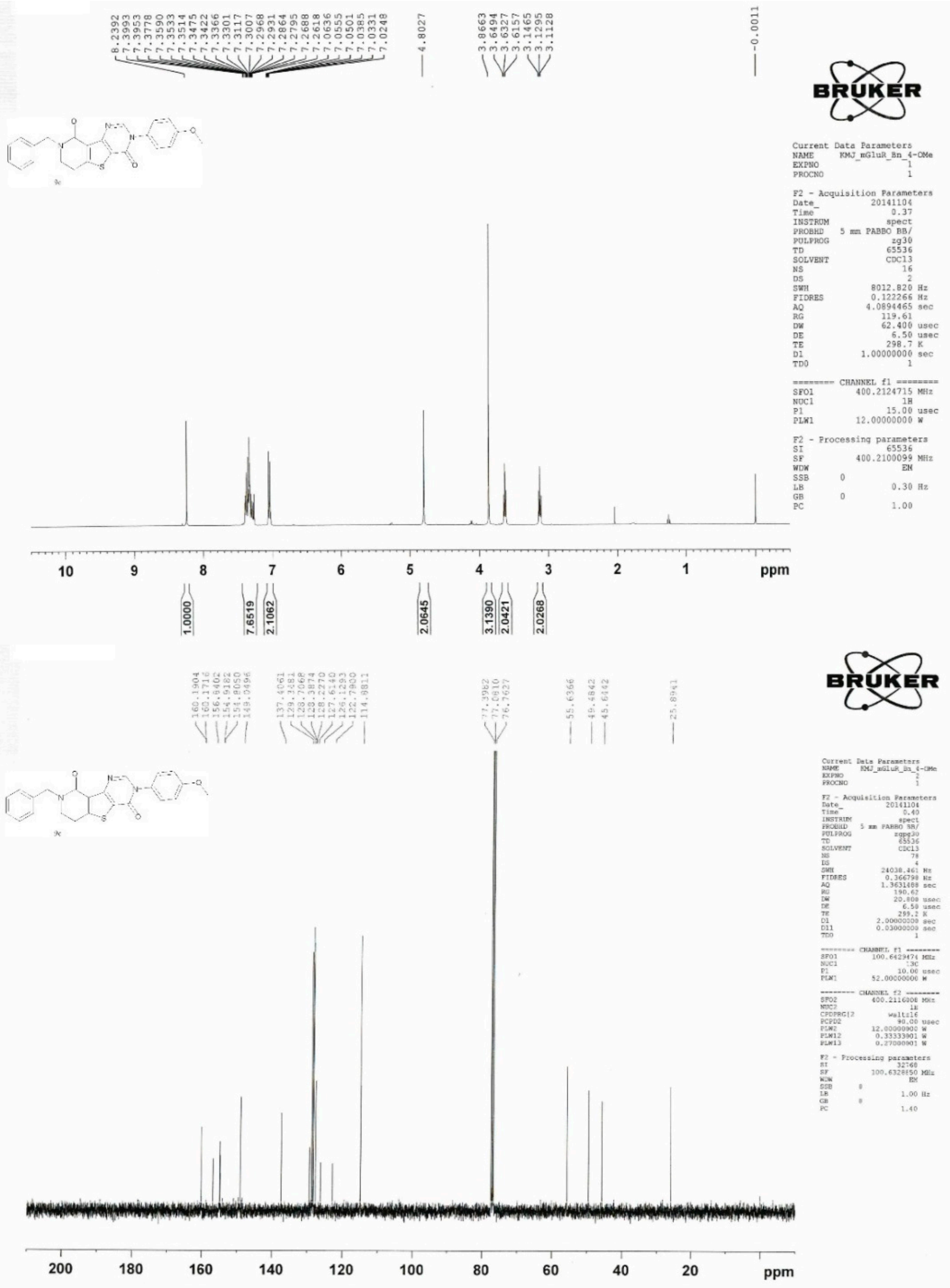

Figure S13. ^{1}H- (upper) and ^{13}C-NMR spectra (lower) of compounds **9c**.

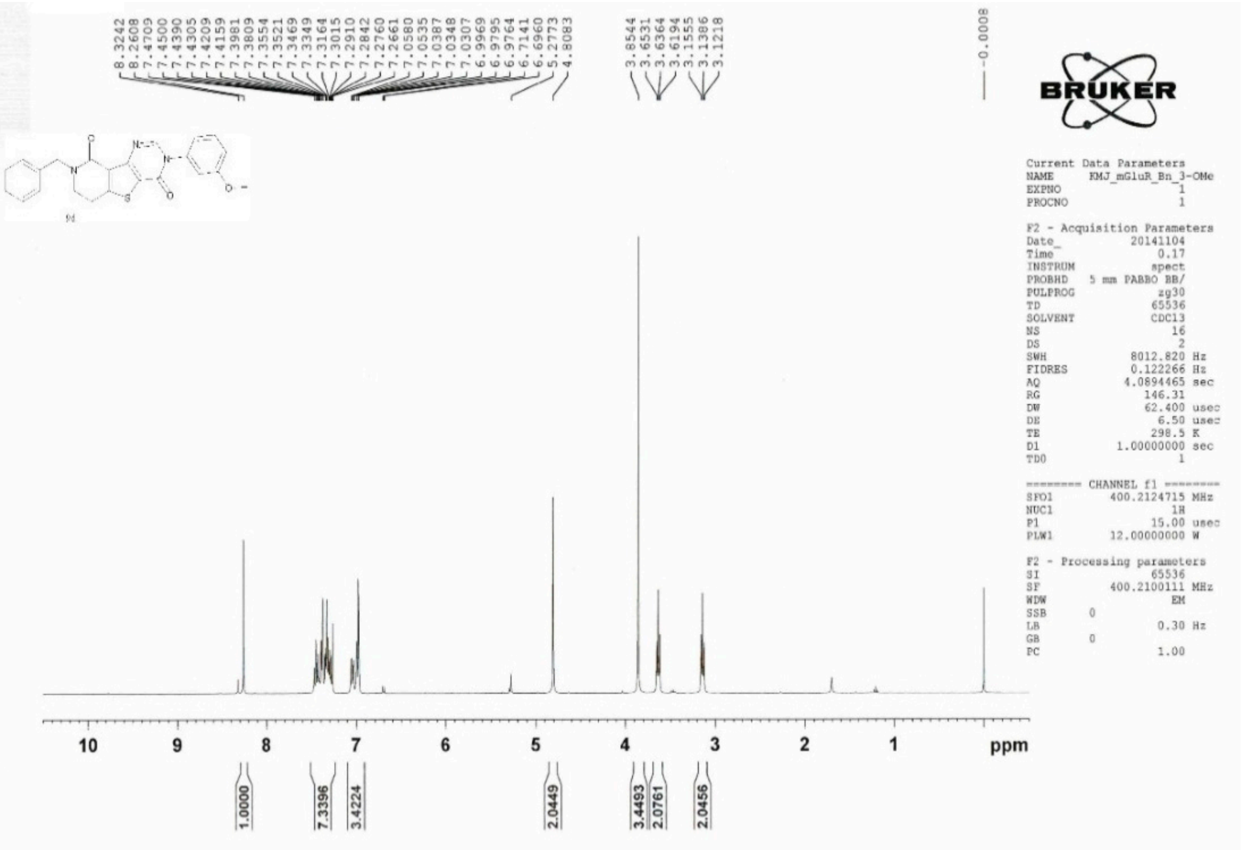

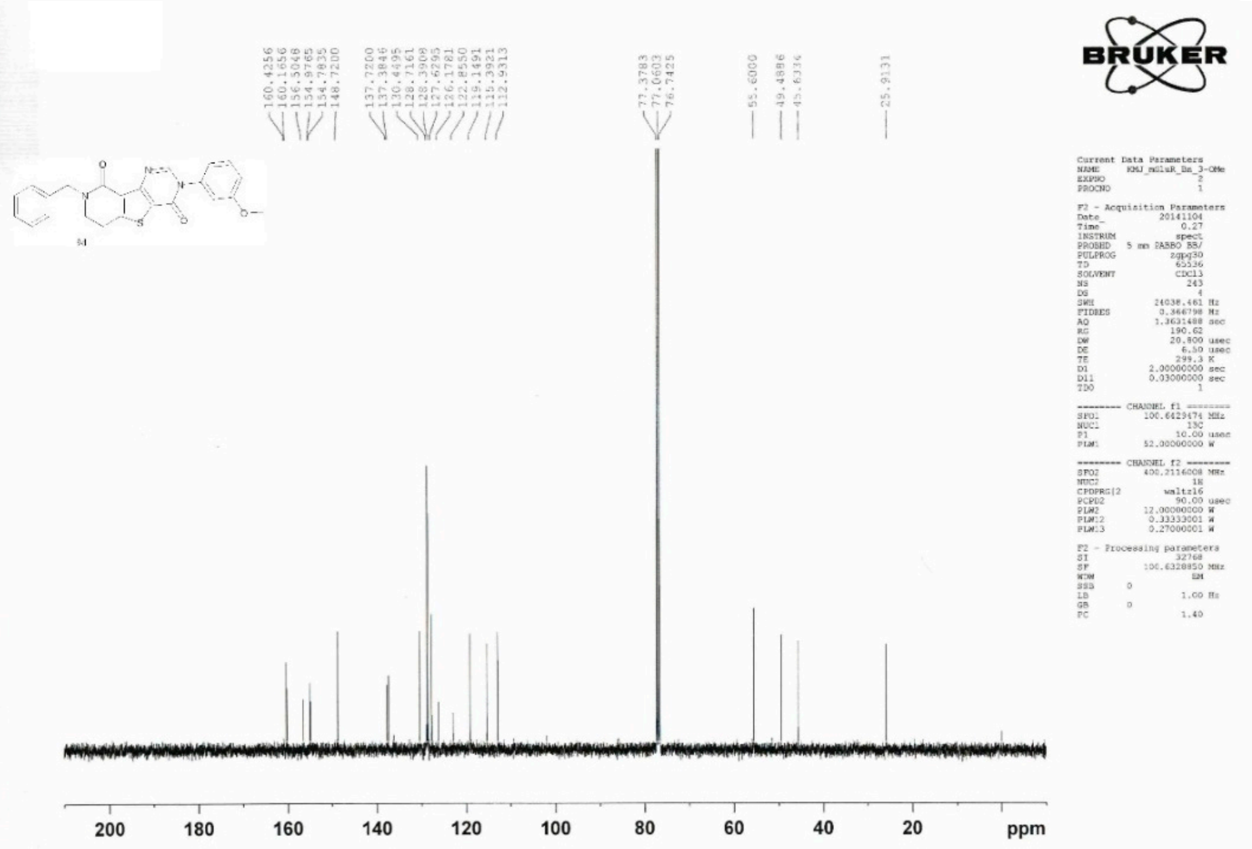

Figure S14. ^{1}H- (upper) and ^{13}C-NMR spectra (lower) of compounds **9d**.

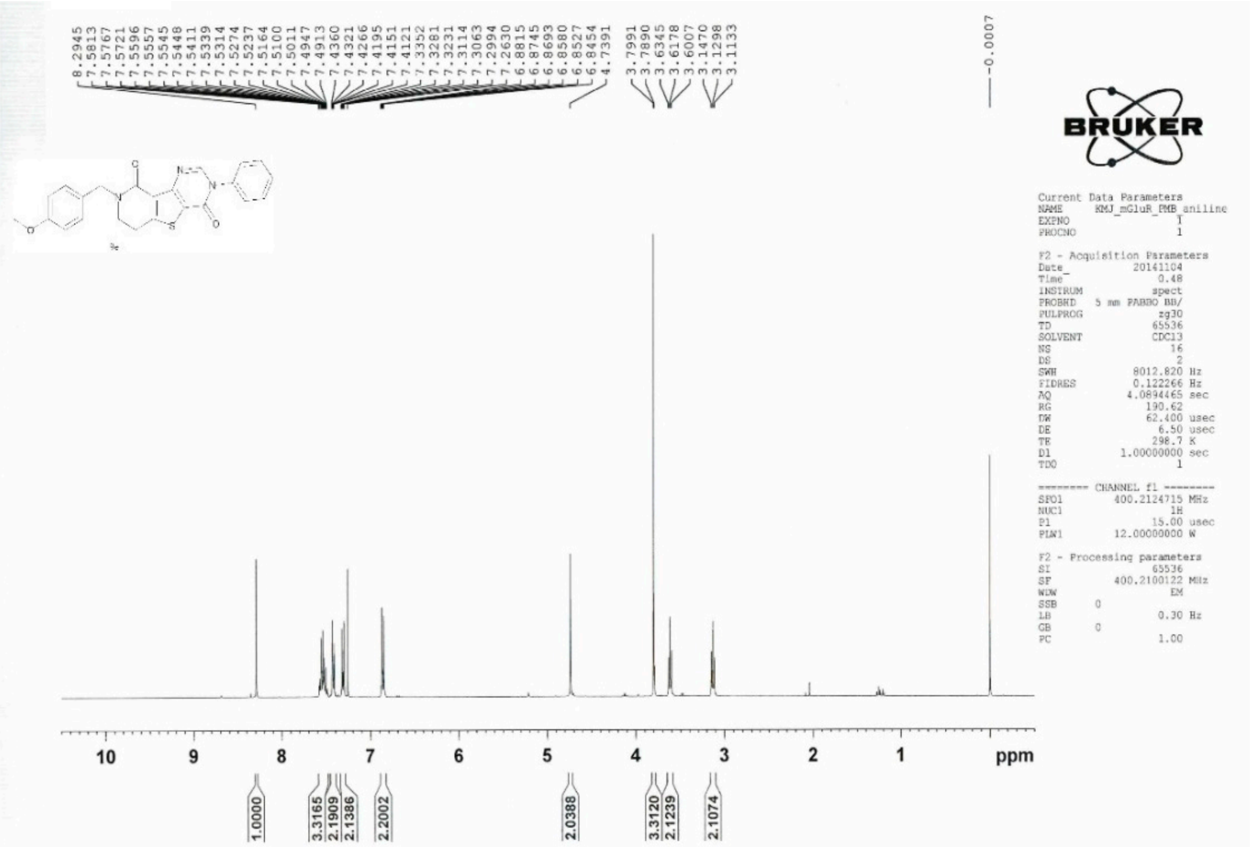

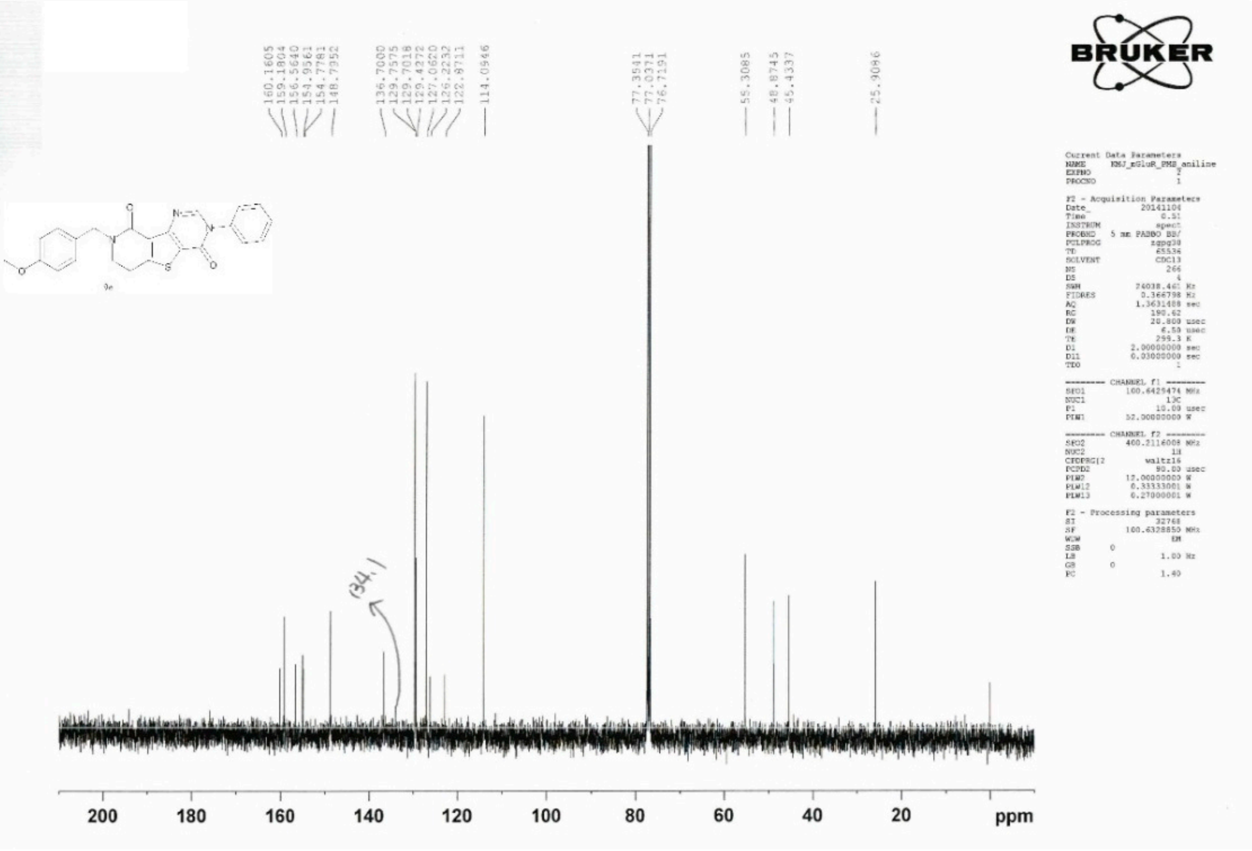

Figure S15. ^{1}H- (upper) and ^{13}C-NMR spectra (lower) of compounds **9e**.

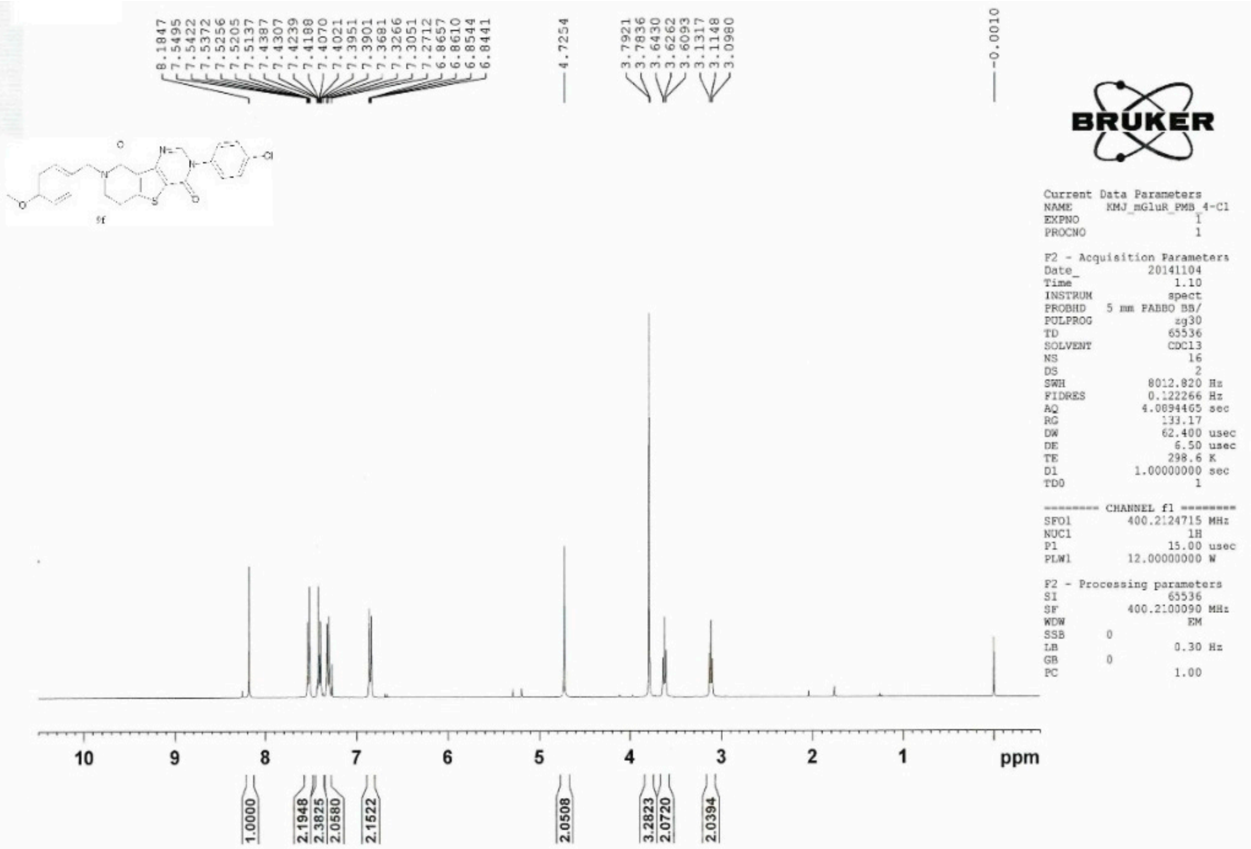

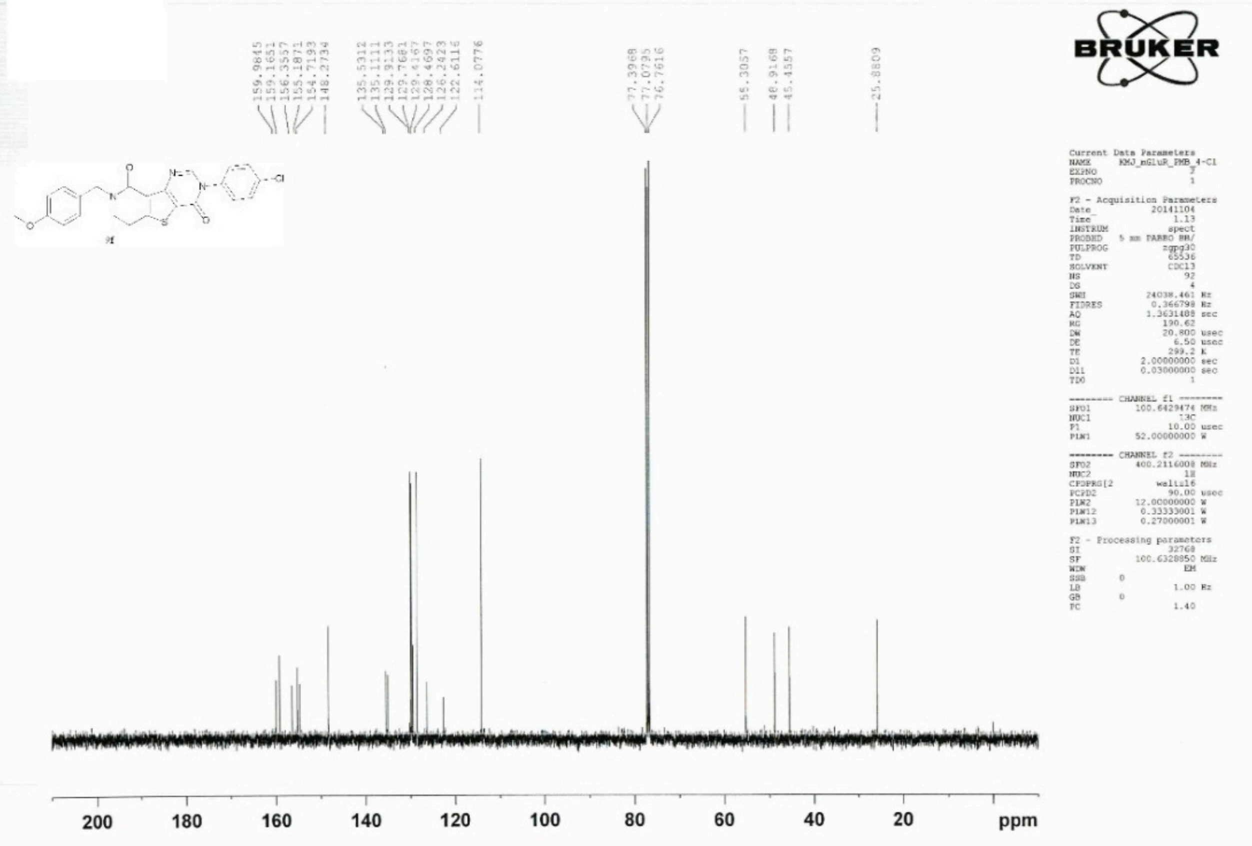

Figure S16. ^{1}H- (upper) and ^{13}C-NMR spectra (lower) of compounds **9f**.

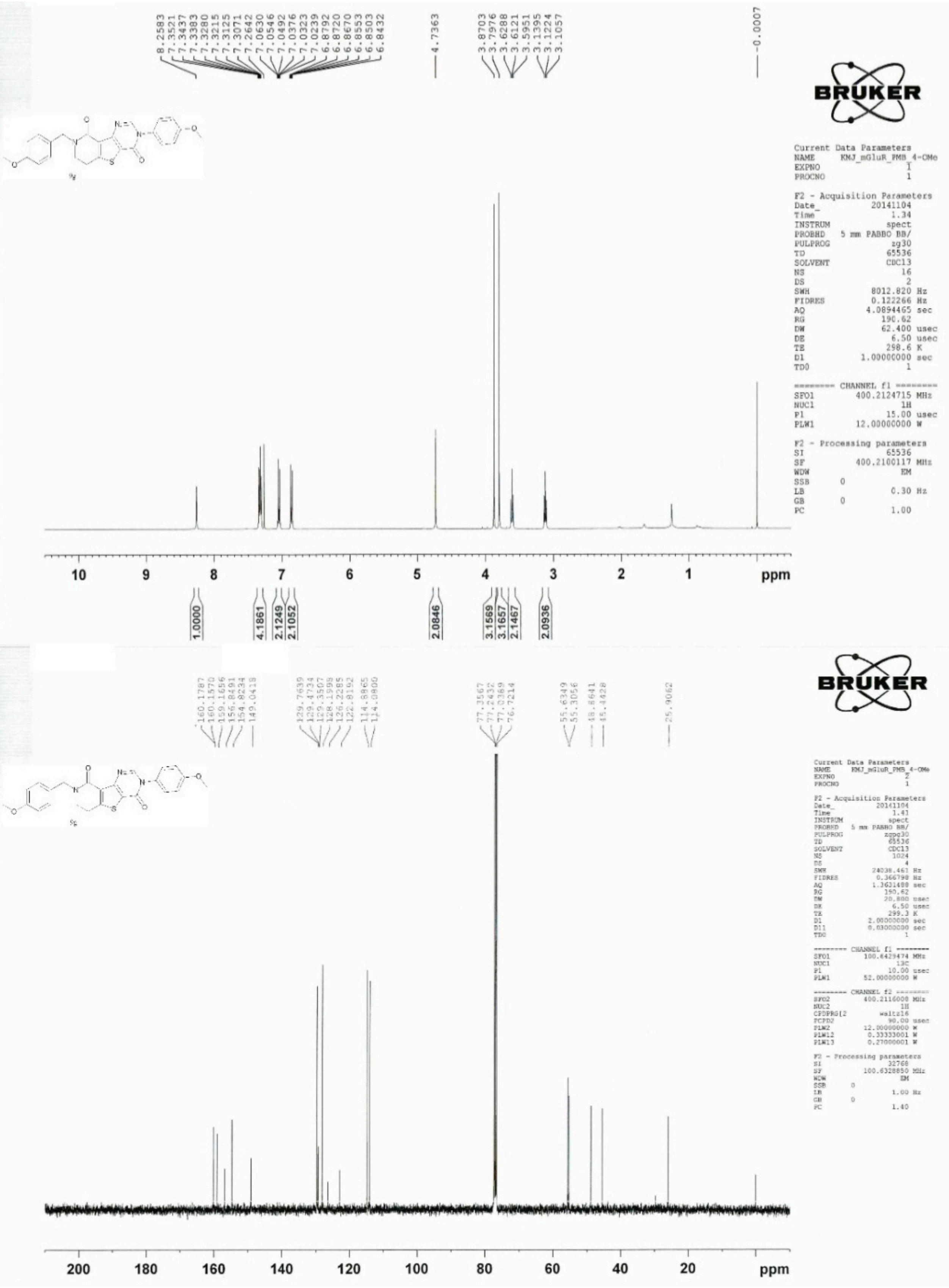

Figure S17. ^{1}H- (upper) and ^{13}C-NMR spectra (lower) of compounds **9g**.

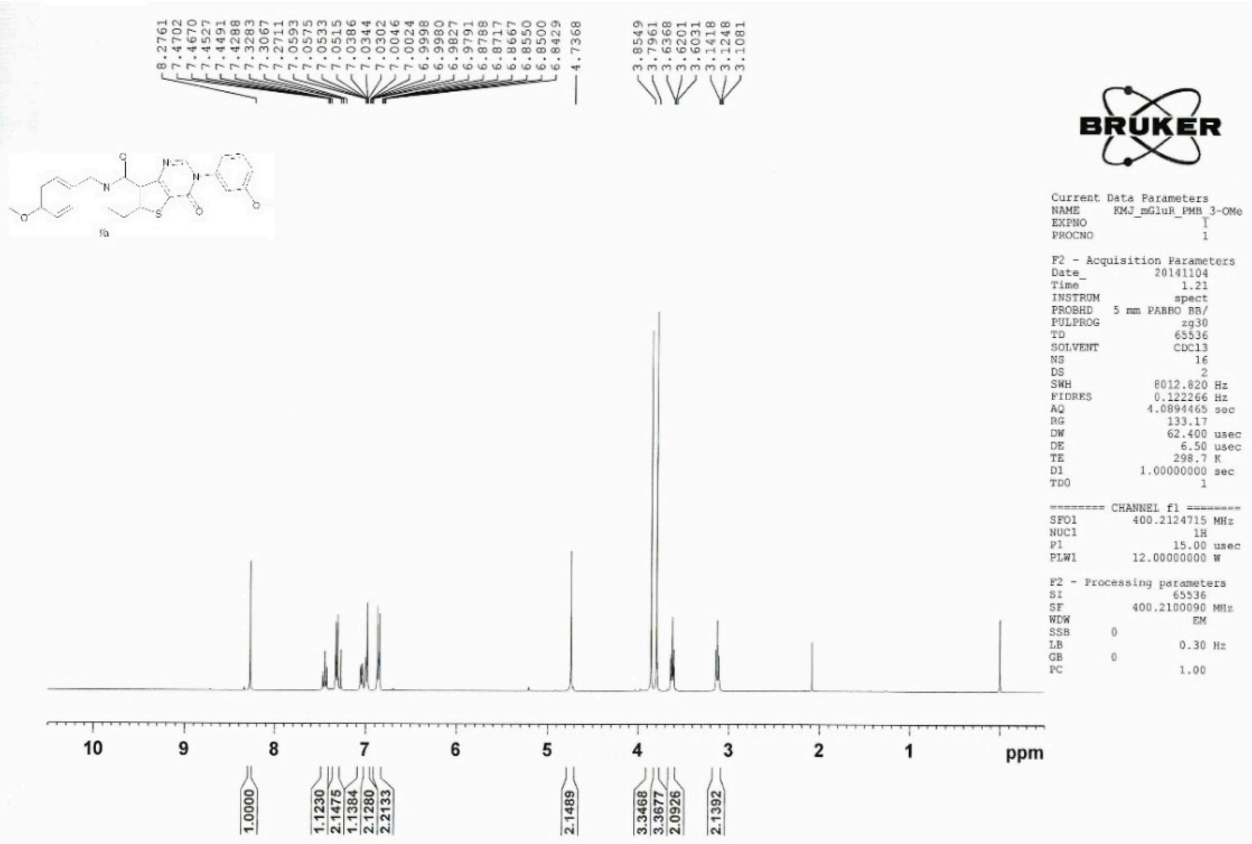

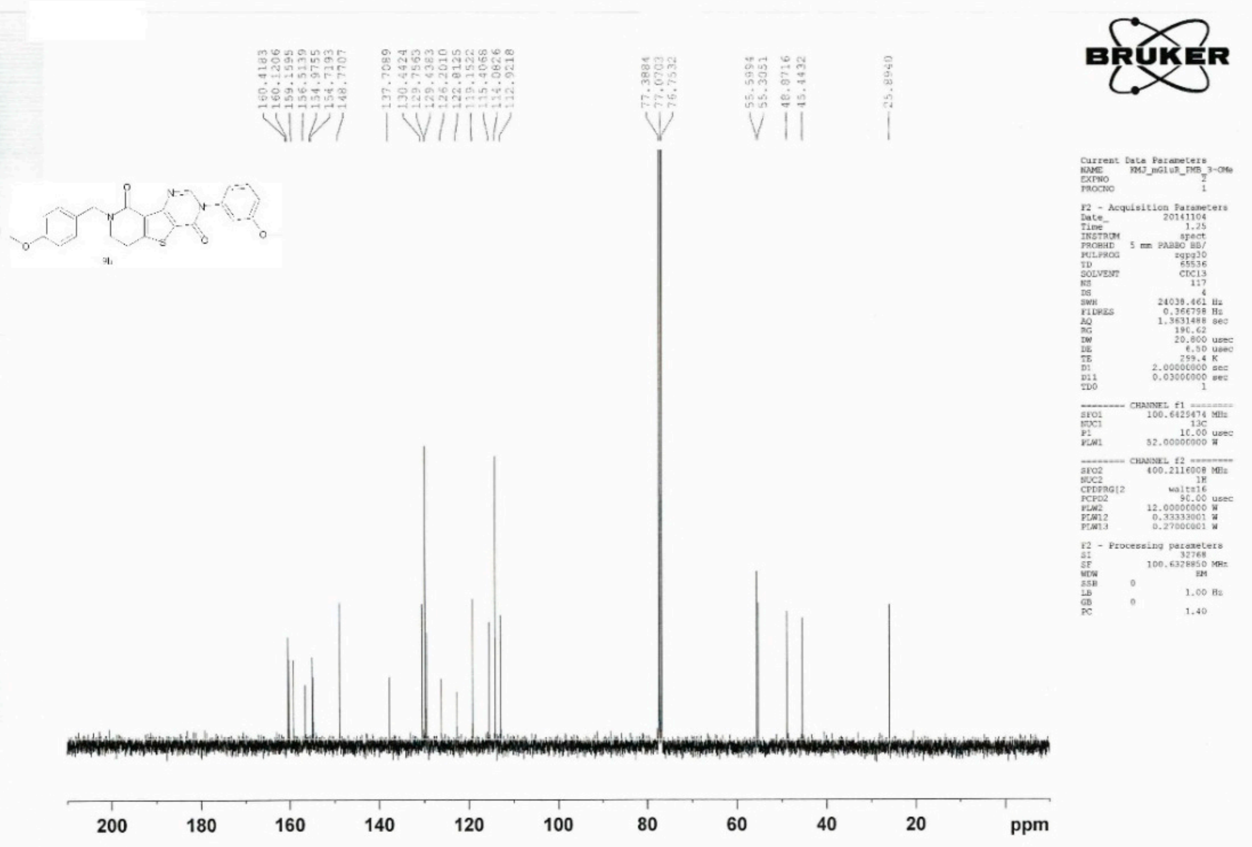

Figure S18. ^{1}H- (upper) and ^{13}C-NMR spectra (lower) of compounds **9h**.
